# ValuesML: A new multilingual dataset for values detection in news and political manifestos

**DOI:** 10.3758/s13428-026-03092-z

**Published:** 2026-08-20

**Authors:** Mario Scharfbillig, Theresa Reitis-Münstermann, Nicolas Stefanovitch, Johannes Kiesel, Paula Schulze Brock, Joanne Sneddon, Emmanuel Cartier, Murat Ardag, Sharon Arieli, Ella Daniel, Henrik Dobewall, Johannes Karl, Anna Krasteva, Thomas Peter Oeschger, Georgios Petasis, Luana Russo, Antonella Seddone, Aurelia Tamo-Larrieux, Hester van Herk, Nazan Avcı, Giuliano Bobba, Pierre Chiron, Ahmet Çoymak, Maria Dagioglou, Dora Katsamori, Ingmar Leijen, Berna Öney, Ricarda Scholz-Kuhn, Emilia Zankina, Sandrine Astor, Petra Auer, Livia Aulino, Anat Bardi, Fiorella Battaglia, Constanze Beierlein, Maya Benish-Weisman, Christina Christodoulou, Patricia R. Collins, Irene Coppola, Mafalda Dâmaso, Meike Morren, Einat Elizarov, Naama Erlich, Peculiar Ezeigwe-Ephraim, Ronald Fischer, Maria Cristina Gaeta, Lucilla Gatt, Noam Gerera, Sjoukje Goldman, Frederic Gonthier, Stefanie Habermann, Mina Hristova, Demet Islambay Yapali, Luigi Izzo, Panos Kapetanakis, Reşit Kışlıoğlu, Chaya Koleva, Roberta Koleva, Joshua Lake, Mael Mesplou, Vanina Ninova, Elif Sandal Önal, Shani Oppenheim-Weller, Duygu Ozturk, Ioannis Elissaios Paparrigopoulos, Vladimir Ponizovskiy, Tim Reeskens, Maria Francesca Romano, Torven Schalk, Alina Starovolsky-Shitrit, Oscar Smallenbroek, Ömer Topuz, Christin-Melanie Vauclair, Giuseppe Di Vetta, Sheng Ye

**Affiliations:** 1 European Commission, Joint Research Centre (JRC), Brussels, Belgium; 2 Arcadia SIT srl, Vigevano, Italy; 3 European Commission, Joint Research Centre (JRC), Ispra, Italy; 4 GESIS – Leibniz Institute for the Social Sciences, Köln, Germany; 5 Bremen International Graduate School of Social Sciences, Bremen, Germany; 6 The Hebrew University Business School, Jerusalem, Israel; 7 Tel Aviv University, Tel Aviv, Israel; 8 Finnish Institute for Health and Welfare, Helsinki, Finland; 9 Dublin City University, Dublin, Ireland; 10 New Bulgarian University, Sofia, Bulgaria; 11 University of Basel, Basel, Switzerland; 12 National Centre for Scientific Research “Demokritos”, Athens, Greece; 13 Maastricht University, Maastricht, The Netherlands; 14 University of Turin, Turin, Italy; 15 The University of Western Australia, Perth, Australia; 16 Vrije Universiteit Amsterdam, Amsterdam, The Netherlands; 17 Middle East Technical University Northern Cyprus Campus, Güzelyurt, Northern Cyprus; 18 Pacte research Centre, School of Political Studies Univ. Grenoble Alpes, Grenoble, France; 19 Abdullah Gül University, Kayseri, Turkey; 20 Carl von Ossietzky Universität Oldenburg, Oldenburg, Germany; 21 University of Basel, Basel, Switzerland; 22 Temple University, Philadelphia, USA; 23 Free University of Bozen-Bolzano, Bolzano, Italy; 24 Università degli Studi Suor Orsola Benincasa, Naples, Italy; 25 Royal Holloway University, London, UK; 26 University of Salento, Lecce, Italy; 27 Ludwig-Maximilians-Universität, München, Germany; 28 Hamm-Lippstadt University of Applied Sciences, Hamm-Lippstadt, Germany; 29 The Hebrew University of Jerusalem, The Paul Baerwald School of Social Work and Social Welfare, Jerusalem, Israel; 30 Edith Cowan University, Joondalup, Australia; 31 Research Centre in European Private Law (ReCEPL), Suor Orsola Benincasa University of Naples, Naples, Italy; 32 Erasmus University Rotterdam, Rotterdam, Netherlands; 33 University of Amsterdam, Amsterdam, The Netherlands; 34 The University of Haifa, Haifa, Israel; 35 Ben-Gurion University Of The Negev, Beersheba, Israel; 36 Iscte-IUL (Instituto Universitário de Lisboa), Centre for Social Research and Intervention, Lisbon, Portugal; 37 IDOR – D’Or Institute for Research and Education, Rio de Janeiro, Brazil; 38 Amsterdam University of Applied Sciences, Amsterdam, The Netherlands; 39 Bulgarian Academy of Sciences, Sofia, Bulgaria; 40 Independent Researcher, Berlin, Germany; 41 Başkent University, Ankara, Turkey; 42 Central European University, Policy and Citizens’ Observatory, Sofia, Bulgaria; 43 Bielefeld University, Bielefeld, Germany; 44 Jerusalem Multidisciplinary College, Jerusalem, Israel; 45 Istanbul Medipol University, Istanbul, Turkey; 46 Ruhr-Universität Bochum, Bochum, Germany; 47 Durham University, Durham, England; 48 Tilburg University, Tillburg, The Netherlands; 49 Scuola Superiore Sant’Anna, Pisa, Italy; 50 Te Herenga Waka - Victoria University of Wellington, Wellington, New Zealand; 51 East China University of Science and Technology, Shanghai, China

**Keywords:** Human values, Value expression, Political discourse, Cross-cultural, Annotation

## Abstract

Values are important building blocks of political ideologies and are frequently invoked in political debates. Yet, because values are abstract, understanding how they are expressed in real-world discourse, and how this varies across contexts, remains a challenge. In this paper, we introduce a large-scale, expert-annotated dataset of value expression in political text, comprising news articles and political manifestos across nine languages. The dataset is grounded in the refined theory of human values (Schwartz et al., 2012) and was developed through an iterative process of annotation guideline development, annotator training, and expert curation. Value annotations capture both the type of value expressed and its evaluative framing, distinguishing whether values are expressed as attained or constrained. The final dataset comprises 2648 texts and 74,231 sentences across nine languages. This resource enables systematic, cross-linguistic analysis of value expression in political communication and provides a benchmark for developing and evaluating computational models of value detection.

**Supplementary Information:**

The online version contains supplementary material available at https://doi.org/10.3758/s13428-026-03092-z.

## Introduction

In democracies, politicians compete for citizens’ votes and approval by offering policy alternatives and persuading voters of their merits (Downs, 1957; Sartori, 1976; Schumpeter, 1976). These processes are shaped by citizens’ systems of values and beliefs, which are rooted in historical and cultural cleavages and, in turn, structure political ideologies and alignments (e.g., Lipset & Rokkan, 1967). A central driver of variation in how individuals respond to political cues is their personal values (Inglehart, 1997; Schwartz, 1992). Values, defined as broad, trans-situational motivational goals (Rokeach, 1973; Schwartz, 1992), are widely regarded as foundational to political ideologies (Feldman, 2003; Jost, 2017; Nelson, 2024; Thorisdottir et al., 2007) and have been shown to predict a wide range of political attitudes and behaviors, including voting (Caprara et al., 2017; Schwartz et al., 2010) and activism (van Herk et al., 2018; Vecchione et al., 2015).

Recent approaches to analyzing value expression in political text have drawn on multiple theoretical frameworks, most notably Schwartz's (1992) theory of human values (e.g., Grigoryan et al., 2023; Ponizovskiy, 2022; Suedfeld et al., 2011) and Moral Foundations Theory (MFT; Graham et al., 2013; e.g., Dillion et al., 2025; Neumann & Rhodes, 2024; Trager et al., 2025). While these frameworks are related, they capture different levels of analysis. Schwartz’s (1992) theory conceptualizes values as abstract motivational goals (e.g., benevolence, security, power) that guide perception and behavior across contexts and are organized in a coherent system of motivational compatibilities and conflicts. In contrast, MFT conceptualizes moral values in terms of domain-specific moral intuitions (e.g., care, fairness, loyalty) that are typically activated in response to perceived moral violations (Graham et al., 2013). Accordingly, moral appeals in political rhetoric, such as references to harm, injustice, or betrayal, can be understood as context-specific instantiations of broader values, aligning with our conceptualization of value expression in text.

This distinction is important for text analysis in the political domain. Approaches based on moral foundations are well suited to identifying moralized language and rhetorical approaches in political communication (e.g., Hopp et al., 2021; Neumann & Rhodes, 2024; Trager et al., 2025), whereas a values-based approach enables the analysis of the underlying motivational structure that organizes such communication. We therefore focus on Schwartz's (1992) theory of human values for three reasons. First, it provides a comprehensive and integrative model of human motivations, extending beyond explicitly moral concerns to include a broader range of goals relevant to political discourse (e.g., power, conformity). Second, its quasi-circumplex structure offers clear theoretical expectations regarding relationships among values, enabling rigorous validity testing (Sagiv & Schwartz, 2022; Schwartz, 1992). Third, Schwartz's theory has demonstrated strong cross-cultural robustness across hundreds of samples in over 80 countries (Schwartz et al., 2012), whereas evidence for the cross-cultural generalizability of MFT is more mixed (Atari et al., 2023; Iurino & Saucier, 2020; Stecker & Hopp, 2026). Taken together, this makes Schwartz’s (1992) theory of human values particularly well suited for comparative, multilingual analysis of value expression in political text.

Despite decades of research on values in the political domain (e.g., Barnea & Schwartz, 1998; Caprara et al., 2006), capturing value expression in text remains challenging due to both the abstract nature of values and context-specificity of political discourse across time and place (Grimmer & Stewart, 2013). Existing approaches to assessing value expression in text have largely relied on dictionary-based methods (see Bardi et al., 2008; Ponizovskiy et al., 2020). These methods depend on word counts, have difficulties capturing contextual meaning (e.g., ambiguity in words such as “party”), and are often limited to specific languages and cultural contexts (i.e., Western, English-speaking populations).

More recently, multilingual transformer-based models have been developed to detect moral values in political text (see Preniqi et al., 2024), improving on dictionary-based approaches (e.g., Simonsen & Widmann, 2025; Widmann & Simonsen, 2025) by capturing contextual meaning and enabling cross-lingual generalization. However, these models are typically trained on task-specific labels and may lack transparency in their training data and annotation procedures, which can limit interpretability and comparability. In contrast, our approach prioritizes theory-driven, expert-annotated data grounded in Schwartz’s (1992) theory of human values, enabling psychologically interpretable and cross-lingually comparable analysis while providing an expert-guided, theoretically grounded resource for training and evaluating computational models for the expression of values in political text.

To address limitations of existing approaches to analyzing value expression in text, we develop a theory-driven dataset of value annotations derived from expert coding of news articles and political manifestos across nine languages (Bulgarian, Dutch, English, French, German, Greek, Hebrew, Italian, and Turkish). By engaging values scholars in the annotation process, the dataset provides an expert-guided, theory-grounded resource for detecting context-sensitive value expression in text. This dataset can be used to investigate how values are expressed in political discourse and to train computational models for cross-lingual value detection (see Kiesel et al., 2024). By combining media coverage and manifestos across diverse political topics, this dataset offers a foundation for analyzing the values–politics nexus and for understanding how policies and political stances are framed in terms of human motivations, with implications for issues such as political polarization and public communication (Smillie & Scharfbillig, 2024).

### The importance of values for understanding political preferences

Values play a central role in guiding human behavior and shaping social systems. They represent enduring, trans-situational beliefs about what is desirable or important and influence preferences, priorities, and actions (Rokeach, 1973; Schwartz, 1992). While individuals share a near-universal set of values, they differ in the relative importance they assign to them, and these differences guide judgments and choices, including political preferences (Schwartz et al., 2010).

**Fig. 1:**
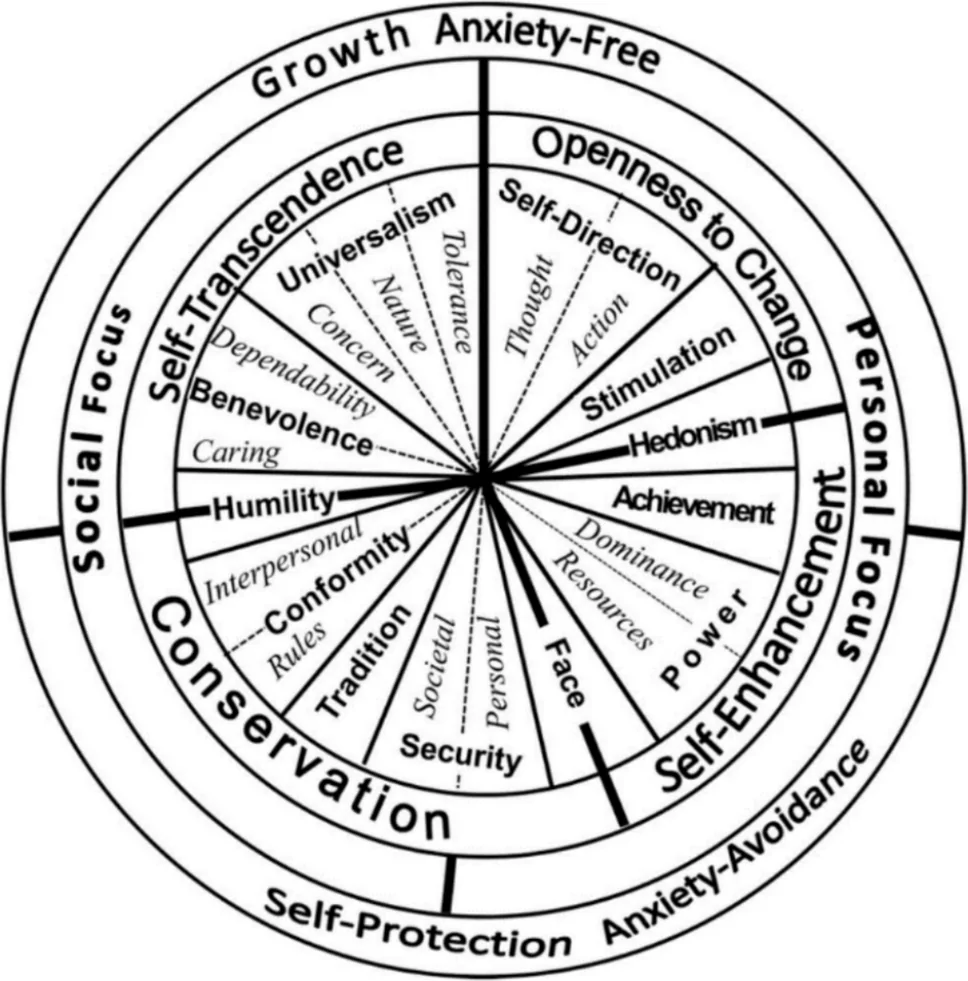
The circumplex structure of Schwartz et al.’s (2012) refined values. *Note*. Schwartz et al. (2012).

Schwartz (1992) developed the most widely cited theory of the content and structure of values in the psychological domain, conceptualizing them as expressions of universal motivational goals derived from basic biological needs, social coordination, and group survival (Schwartz & Bilsky, 1987). In his theory, Schwartz identified ten basic values that express distinct motivations (i.e., power, achievement, hedonism, stimulation, self-direction, universalism, benevolence, tradition, conformity, and security). These values are organized in a quasi-circumplex structure around a continuum of motivational conflicts and compatibilities (see Fig. 1, inner circle in bold text) and can be summarized into two higher-order value dimensions: self-transcendence versus self-enhancement, and conservation versus openness to change.

Subsequent research further partitioned the circumplex into 19 conceptually distinct refined values (Schwartz et al., 2012; see Fig. 1 inner circle). Consistent with Schwartz’s (1992) definition of values as abstract and universal, the 19 refined values have been shown to hold similar meanings across cultures (Cieciuch et al., 2014; Schwartz & Cieciuch, 2022).

While basic and refined values themselves are universal, their behavioral expressions are context dependent. For example, although environmental protection is widely valued, people in different countries associate it with different actions (e.g., conserving water in Brazil vs. recycling in the UK; Hanel et al., 2018a, b). Such variation is particularly pronounced in politics (Caspers et al., 2011), where shared values can underpin divergent interpretations of the same policy. Despite broad overlap in value priorities across political groups (Wolf & Hanel, 2024), policies may be interpreted as humanitarian by some and harmful by others, a dynamic amplified in contemporary polarized social media environments (Van Bavel et al., 2024; Scharfbillig et al. 2026).

Recent theoretical work conceptualizes values as abstract cognitive categories that are expressed through context-specific instantiations (i.e., concrete actions, events, or policies that reflect underlying motivational goals; Maio, 2010). This perspective reconciles the universality of values with the variability of their expression: while values are broadly shared, their instantiations differ across cultural, social, and historical contexts. Empirical research supports this view, showing systematic variation in value instantiations across cultures and political groups (Hanel et al., 2018a, b; Ponizovskiy, 2022). The idea that people perceive the same actions, events, and policies through different values and moral principles has informed the development of effective communication strategies, particularly for bridging political divides (e.g., Feinberg & Willer, 2015), challenging earlier findings that persuasive communication requires alignment of abstract values (e.g., Maio et al., 2014).

However, research on value instantiations, particularly in political contexts, remains limited, especially in cross-cultural settings (e.g., Macdonald, 2023; Scharfbillig et al., 2025). This project addresses this gap by introducing a multilingual, cross-cultural dataset that links value expression in text to specific policies and events, enabling fine-grained analysis of value instantiations in political discourse.

### Current approaches to measuring values in text

Research on values in text has largely relied on dictionary-based approaches to detect value expression (e.g., Bardi et al., 2008; Ponizovskiy et al., 2020; Suedfeld et al., 2011; Suedfeld & Brcic, 2011). These methods identify values through predefined word lists and have been applied to diverse domains, including political speeches, public discourse, and policy debates. However, dictionary-based approaches are limited in their ability to capture contextual meaning, as they assume stable word-value associations and struggle with ambiguity (Stefanovitch et al., 2023). This limitation extends to probabilistic dictionaries, which weight words based on co-occurrence patterns, but still rely on fixed word-to-value relations. Such assumptions may be especially problematic in ideologically loaded contexts, where the same words can convey different values depending on usage (e.g., “freedom of” vs “freedom to”; Passini, 2017).

In contrast to standard and probabilistic dictionaries, natural language processing (NLP) approaches enable a more context-sensitive analysis of value expression by leveraging co-occurrence patterns and contextual representations. In particular, transformer-based models can capture meaning beyond individual words and have been used to train classifiers for values and moral content (Devlin et al., 2019). For example, Preniqi et al. (2024) demonstrate that such models outperform dictionary-based approaches, highlighting the importance of contextualization in detecting normative content. This aligns with evidence that differences in political communication often lie not in what values are expressed, but in how they are framed (Kraft & Klemmensen, 2024).

Despite these advances, several limitations remain. Transformer- and LLM-based approaches are typically trained on task-specific labels and may lack transparency in training data and annotation procedures. Further, while LLM-generated or weakly supervised corpora are increasingly used, prior work shows that such approaches remain dependent on high-quality human-annotated data for calibration and evaluation and do not yet provide a full substitute for gold-standard annotation (Devlin et al., 2019; Ratner et al., 2017; see also Trager et al., 2025; Zangari et al., 2025). In addition, existing NLP research frequently lacks clear and consistent theoretical grounding, with varying conceptualizations of moral and value-related constructs, limiting interpretability and comparability across studies (Zangari et al., 2025; see also Vida et al., 2023).

Although NLP approaches to moral content have advanced rapidly, work on human values remains limited. Recent efforts to address this gap include annotated datasets of value expression in argumentative texts (Kiesel et al., 2022, 2023). In this work, crowd workers annotated short justifications for policy positions in a debate corpus using Schwartz et al.’s (2012) 19 refined values.^1^ While this dataset enables the detection of value expression in structured arguments, it is restricted to narrowly defined argumentative contexts and does not generalize to broader forms of political discourse. This is a key limitation, as arguments constitute only a subset of political communication (e.g., news and speeches).

To address this gap, the present study introduces a multilingual, cross-cultural dataset of expert-guided value annotations in political news and manifestos. By capturing value expression in more complex and widely occurring forms of political text, this dataset enables a more comprehensive analysis of how values are expressed in real-world political discourse.

### The value annotations dataset

The value annotations dataset presented in this paper offers several features that make it a valuable resource for values research, political analysis, and computational social science. First, to the best of our knowledge, it is the largest available dataset of annotated value expressions in text. Second, it includes annotations in nine languages (i.e., Bulgarian, Dutch, English, French, German, Greek, Hebrew, Italian, and Turkish), spanning diverse cultural contexts. This multilingual scope extends beyond the predominantly English-language focus of prior work (e.g., Ponizovskiy, 2022; Suedfeld et al., 2010, 2011) and enables cross-cultural comparisons of value expression, complementing findings from survey research (Beugelsdijk & Welzel, 2018; Inglehart, 1990). To ensure consistency across languages, annotators in all language groups undertook the same training and followed the same annotation protocol (see Reitis-Münstermann et al., 2024).

Third, the dataset covers a broad range of politically salient topics, including energy, economy and industry, education and science, environment and climate change, health (including COVID-19), immigration, crime and justice, national security, European Union and international relations, social care and human rights, and working conditions. This topical diversity makes the dataset well suited for analyzing political discourse and societal conflicts.

Fourth, annotations were produced by experts in values research, drawn from social psychology, political science, sociology, business and economics, and data science, to ensure close alignment with Schwartz's (1992) theory of human values. This approach reflects the interpretive complexity of value identification: although expert coders typically achieve only modestly higher inter-annotator agreement than non-experts (≈.10–.11 *α*), they provide more consistent and theoretically grounded judgments, which is critical for construct validity (Milkova et al., 2023). This contrasts with prior datasets relying on crowd-sourced annotations (e.g., Kiesel et al., 2022, 2023) and supports a theory-driven approach to value annotation.

Fifth, the dataset includes text-span-level annotations that capture the contextualized expression of values, moving beyond word-level dictionary approaches (e.g., Bardi et al., 2008; Ponizovskiy et al., 2020). This allows values to be interpreted in context, accounting for ambiguity and variation in meaning across different usages.

Finally, the dataset introduces a second annotation layer capturing value fulfillment, distinguishing whether a value is expressed as attained, constrained, or ambiguous. This distinction reflects the dual nature of values as both desirable and aspirational (Rokeach, 1973, Schwartz, 1992; Woltin & Bardi, 2018). In political discourse, values may be invoked as being promoted or attained (e.g., a policy framed as ensuring freedom and democracy, promoting the attainment of universalism values) or as being threatened or constrained (e.g., the same policy framed as threatening our safety, thwarting security values). The dataset captures this directionality by coding whether a value is expressed as (partially) attained, (partially) constrained, or ambiguous, enabling a more contextual analysis of how values are framed and contested in political text.^2^

## Method

In this section, we describe the corpus construction, annotator recruitment and training, and the annotation and curation procedures. The project was conducted from May 2023 to April 2024 and coordinated by a core project team at the Joint Research Centre (JRC) of the European Commission. This team designed and managed the annotation campaign, recruited expert annotators, and developed the annotation guidelines.

Annotation teams were led by language leads, who received onboarding and training from the core team. Regular meetings were held with language leads to review example annotations and resolve ambiguities in value classification. Throughout the project, the core team monitored annotation progress and provided ongoing technical and procedural support.

### Participants and procedures

We recruited values experts to fill the roles of annotators, curators, and language group leads in the annotation process, via (a) an open call issued by the JRC and (b) snowball sampling through existing research networks. The decision to recruit experts was motivated by the interpretive complexity of value identification and the need to ensure close alignment between annotations of value expression and Schwartz’s (1992) theoretical framework.

Eligibility criteria required participants to have research experience in human and/or cultural values and high proficiency in at least one of the nine focal languages. Participants were assigned to language-specific groups and allocated roles as annotators, curators, and/or language leads. In total, the annotation campaign involved 66 annotators, 21 curators, and nine language leads.^3^

Annotators were responsible for reading assigned texts and identifying and annotating spans of text for value expression. The number of texts per annotator varied depending on availability and language-specific needs. Curators, selected based on their extensive expertise in values research, reviewed annotated texts and resolved disagreements. Each language group was coordinated by one or more language lead, who oversaw recruitment, onboarding, and training, and managed the allocation of texts for annotation and curation.

### Materials

The corpus comprised texts from two primary sources: (1) news articles extracted from the Europe Media Monitor (EMM)^4^ and (2) excerpts from manifestos of major political parties in the focal languages.^5^ The procedures for selecting and processing texts from each source are described in detail below.

#### News media

The selection of news articles followed a two-stage process: (1) automated pre-selection and (2) manual selection and cleaning.

##### Automated pre-selection

For the European Union language groups (Bulgarian, Dutch, English, French, German, Greek, and Italian), we first drew a random sample of 32,500 news articles per language (400–800 words in length) from the EMM, covering both mainstream^6^ and unverified sources.^7^ Articles were published between January 1, 2019, and March 31, 2023. From this pool, we retained articles tagged with the following EMM topic categories:^8^ Immigration/refugees, working conditions, economy/industry, environment/energy, health, defense, and education/role of science. For each topic, we randomly selected 75 articles from mainstream sources and ten from unverified sources, resulting in approximately 600 articles per language.

For Turkish and Hebrew, we initially retrieved 510 articles (400–800 words in length) from all EMM sources and 150 from unverified sources. As many Hebrew articles were excluded during screening due to a lack of relevance to the focal topics, an additional 350 articles (plus 90 articles from unverified sources) were retrieved. Unlike the EU languages, these samples were not pre-stratified by topic. For Turkish, an additional semi-supervised topic modeling step was used to classify article content prior to manual selection.

##### Manual selection and cleaning

The pre-selected articles were provided to language leads in spreadsheet format (see Annex 2). Language leads selected up to 300 articles that were deemed “value-laden” (i.e., likely to contain value expression), while maintaining a balanced distribution across topics and source types (mainstream vs unverified).^9^

This step reflects a signal-driven sampling strategy, designed to increase the density of value-relevant content and ensure the efficient use of expert annotation resources. Value expression in naturalistic text is relatively sparse, with prior work suggesting that only around one-third of texts contain identifiable value content (Milkova et al., 2023). Consequently, purely random sampling would yield a large proportion of articles with little or no annotatable signal (e.g., descriptive reports on financial markets or sports results), reducing both efficiency and data quality. The resulting dataset is therefore not intended to be representative of the full distribution of news content, but rather to provide a theoretically informative sample in which value expression is sufficiently prevalent to be reliably identified and analyzed. Importantly, this preselection operates at the level of stimulus sampling and does not determine what counts as a value; value identification remains grounded in theory and is conducted during annotation.

Initial screening was based on brief article excerpts, followed by verification or reassignment of topic categories using a predefined list or a free-text option. Free-text entries were subsequently harmonized across languages to ensure consistency in topic classification (see Table 1, column 1). Selected articles were then cleaned by removing duplicates, symbols, URLs, and copyright information.^10^

**Table 1:** Total news article counts per topic for each language at the curation stage

	Bulgarian	German	Greek	English	French	Hebrew	Italian	Dutch	Turkish	Total	Mean	SD
(Renewable) Energy	14	1	12	4	4	4	16	14	88	157	17.4	27.0
Economy/industry	42	44	51	44	39	53	82	59	35	449	49.9	14.1
Education/role of science	10	7	11	18	11	12	11	10	48	138	15.3	12.6
Environment/climate change	4	17	11	19	10	4	6	21	0	92	11.5	6.8
Health/COVID	16	22	26	61	22	36	44	42	39	308	34.2	14.1
Immigration/refugees	6	8	18	8	13	3	28	22	18	124	13.8	8.3
Judiciary/crime	9	14	5	32	4	10	0	14	0	88	12.6	9.4
National defense/security	10	25	37	41	12	47	31	44	31	278	30.9	13.2
Other	9	30	10	40	10	6	0	8	0	113	16.1	13.3
Politics	49	33	55	51	31	16	0	7	0	242	34.6	18.3
Role of EU/international relations	33	2	49	11	10	11	13	23	14	166	18.4	14.4
Social care/human rights	9	6	6	29	14	8	0	6	0	78	11.1	8.4
Working conditions/minimum wage	15	12	7	15	5	4	14	23	26	121	13.4	7.6
Total	226	221	298	373	185	214	245	293	299	2354		
Mean	17.4	17.0	22.9	28.7	14.2	16.5	27.2	22.5	37.4	181.1		
SD	14.4	13.1	18.6	17.8	10.5	17.2	23.8	16.3	23.2	108.9		

As shown in Table 1, the final set of news articles selected for annotation comprised 2354 texts. The number of articles per topic ranged from 78 (social care/human rights) to 449 (economy/industry). Across language groups, article counts ranged from 185 (French) to 373 (English).

The corpus was designed to capture value expression in political texts, rather than to provide uniform coverage across all topic domains. Accordingly, variation in topic distributions across languages reflects differences in media coverage within the EMM system and language-specific filtering decisions (e.g., Hebrew resampling), rather than a theoretically driven imbalance. Given the focus on political discourse, the primary criterion was ensuring sufficient coverage of value-relevant content within these domains, rather than achieving equal representation across all topic categories.

#### Political manifestos

To capture a diverse range of political perspectives, we selected manifestos from 70 political parties that competed in the most recent parliamentary elections^11^ (2019–2023) in Austria, Germany, Bulgaria, France, Greece, Italy, the Netherlands, Turkey, Israel, Ireland, the United Kingdom, and the USA. Parties were primarily selected based on parliamentary representation (i.e., number of seats in parliament), with additional parties included in countries with fewer than six parliamentary parties based on vote share.

For each party, we extracted manifesto excerpts related to key policy areas: (renewable) energy, immigration/refugees, minimum wage, national defense/security, education, and economy/industry. The Greek language group additionally included excerpts classified as “international affairs” and “most topics” where content spanned more than one pre-defined category.

As shown in Table 2, the dataset includes 294 manifesto excerpts (*M* = 32.7 per language group). The number of excerpts per topic ranged from 37 (minimum wage) to 58 (economy/industry). Across language groups, excerpt counts ranged from 24 (Turkish) to 36 (Hebrew).

**Table 2:** Total manifesto excerpt count per topic for each language

	Bulgarian	German	Greek	English	French	Hebrew	Italian	Dutch	Turkish	Total	Mean	SD
(Renewable) Energy	4	7	3	5	6	5	5	5	4	44	4.9	1.2
Economy/industry	8	9	5	8	6	7	6	5	4	58	6.4	1.7
Education	7	6	4	5	6	6	5	5	4	48	5.3	1.0
Immigration/refugees	3	8	4	6	6	7	6	5	4	49	5.4	1.6
Mixed topics	0	0	5	0	0	0	0	0	0	5	0.6	1.7
National defense/security	7	8	3	6	6	6	6	5	4	51	5.7	1.5
International affairs	0	0	2	0	0	0	0	0	0	2	0.2	0.7
Minimum wage	5	2	4	5	4	5	3	5	4	37	4.1	1.1
Total	34	40	30	35	34	36	31	30	24	294	32.7	4.6
Mean	4.3	5.0	3.8	4.4	4.3	4.5	3.9	3.8	3.0	36.8		
SD	3.1	3.7	1.0	2.9	2.7	2.9	2.6	2.3	1.9	21.4		
Number of political parties	7	8	7	13	6	8	6	11	4	70		

### Annotations

#### Annotator training

The annotator training was designed to promote rapid skill development and a shared understanding of the expression of Schwartz et al.’s (2012) refined values in political texts across language groups. Annotators were trained to (1) identify value-laden spans of text, (2) assign the relevant refined value(s) expressed in each span, and (3) indicate whether the value was expressed as attained, constrained, or undecided. To support consistency, annotator training emphasized prioritizing the evaluative meaning most directly expressed in the text. Explicit normative content was prioritized over implied meaning, while more distal or instrumental interpretations were treated as secondary. Although multiple values could be assigned to the same sentence or overlapping text spans when clearly present, annotators were encouraged during training to identify a dominant value in ambiguous cases to enhance reliability and reduce over-attribution.

Training proceeded in three stages. First, all annotators and curators were introduced to a comprehensive annotation guide (Reitis-Münstermann et al., 2024) by their language leads. The guide outlines Schwartz et al.’s (2012) theory of refined values and provides detailed instructions for identifying and annotating the expression of the 19 refined values in text. Although an initial version of the guide was used at the outset, it was iteratively updated throughout the project based on feedback and the resolution of ambiguous cases at both the language group and project levels.

Second, participants completed a structured training program using an online platform with flashcard-based exercises. Each flashcard presented a sentence alongside a set of candidate values and required annotators to identify the dominant value(s) and indicate whether the value was expressed as being attained or constrained (see Supplemental Materials, Annex 1). The flashcards were developed iteratively from value-laden sentences drawn from English-language news articles and were refined through bi-weekly discussions with the core project team and language leads. During this process, candidate annotations were compared, disagreements were resolved through discussion, and examples were revised or excluded where no clear resolution could be reached. This iterative approach served as a form of content validation, ensuring that training items closely reflected annotation guidelines and supported the development of shared decision rules.

Flashcards were organized into four levels of increasing difficulty, progressing from relatively unambiguous sentences with a limited set of candidate values (level 1) to more complex and context-dependent cases with a broader range of value options (level 4).^12^ The level 4 flashcards often involved interpretive challenges, and these cases were used as training opportunities to develop shared decision rules, with an emphasis on identifying the dominant value expressed in context. For example, when multiple values could plausibly be inferred from a sentence, annotators were instructed to prioritize the value most directly and explicitly expressed in the text, rather than more distal or instrumental interpretations. At the same time, annotators were permitted to assign multiple values, including overlapping spans, when distinct value meanings were clearly present.

Third, prior to the main annotation phase, all annotators completed a calibration task by annotating the same political speech using the annotation platform.^13^ These annotations were reviewed by language leads and discussed within language groups to assess agreement and further align annotation practices.

#### The annotation campaign

The annotation campaign ran from May 2023 to April 2024. Throughout this period, language leads met weekly with the core project team via MS Teams to discuss complex annotation cases and agree on consistent solutions (see Supplemental Materials, Annex 3). Prior to each meeting, language leads submitted challenging cases to a shared online spreadsheet. Agreed resolutions were incorporated into the annotation guidelines and communicated to all annotators within each language group. All meetings were recorded and made available to the core project team and language leads.

Approximately 4 months into the annotation campaign, once sufficient data had been annotated, inter-annotator agreement (IAA) was assessed. Pairwise agreement within each language group was calculated using Cohen’s kappa, while overall agreement for each language was estimated using Krippendorff’s α (Krippendorff, 2018). These metrics were reviewed and discussed during project meetings to identify areas requiring further training and alignment.

In addition, language-specific meetings were held fortnightly and led by language leads. These sessions provided a forum for annotators to raise complex cases, discuss guideline updates, and review IAA results, supporting ongoing calibration and consistency in annotation practices.

#### The annotation guidelines

**Fig. 2:**
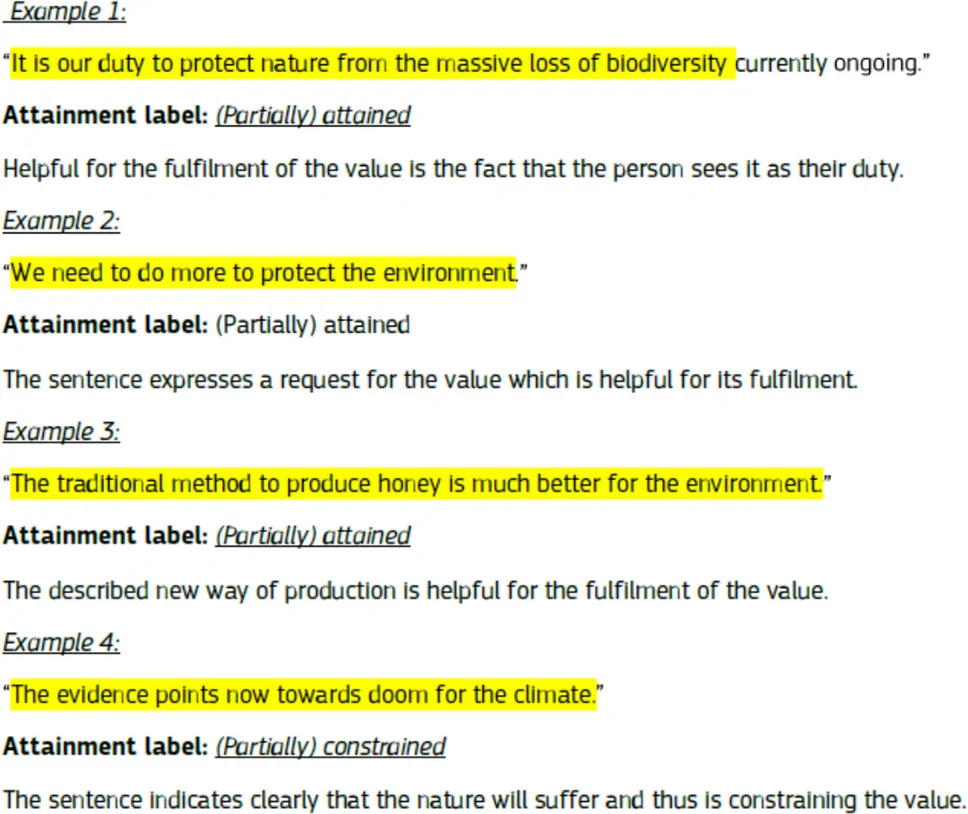
Example annotation of the expression of universalism-nature in text. *Note*. Reitis-Münstermann, et al. (2024)

Annotators were instructed to (1) identify value-laden spans of text, (2) assign the relevant refined value(s) expressed in the text span, and (3) indicate the attainment level of the assigned value(s) (i.e., attained, constrained, or undecided). Figure 2 provides an example from the annotation guide (Reitis-Münstermann et al., 2024), illustrating the annotation of a text span expressing universalism-nature, along with its attainment label.

Importantly, the annotation framework allows for multiple values to be expressed within the same sentence or text span. Annotators could assign more than one value label, including overlapping spans, when distinct value expressions were clearly present. However, consistent with the training protocol, emphasis in the annotation guidelines was on identifying the dominant value in context to promote reliability and reduce over-attribution in ambiguous cases. In line with the annotation guidelines, annotators were instructed to prioritize the value most directly and explicitly expressed in the text, rather than more distal or instrumental interpretations. This principle was reinforced in flashcard training, where complex examples were used to develop shared decision rules while still acknowledging that value expressions can be multifaceted.

Annotations were conducted using the INCEpTION platform (Klie et al., 2018), a web-based interface designed for collaborative annotation projects.^14^ Annotators accessed assigned texts within the platform and could adjust the number of visible sentences to incorporate broader context, including titles. Value annotations were applied by highlighting value-expressive text spans and assigning corresponding value and attainment labels.

Annotators were encouraged to select text spans up to the sentence level to preserve contextual meaning, but could annotate longer or shorter spans if justified (e.g., when consecutive sentences formed a coherent argument). Overlapping annotations were permitted where multiple values were expressed within the same text span. In total, 43,248 text spans were annotated.

Table 3 summarizes annotator participation and annotation output by language group. Annotators completed an average of 41 texts each, ranging from 28 (Hebrew) to 81 (Dutch). Most texts (82%) were annotated by two experts, while 12% were annotated by a single expert. Thus, while most texts received dual annotations, reliability was further supported through shared training, overlapping assignments, and ongoing calibration procedures, enabling consistency at the dataset level rather than relying solely on per-item agreement. Single-annotator cases primarily arose due to resource constraints and were retained to preserve corpus coverage.

**Table 3:** Number of annotators, texts, and sentences by language group at the curation stage

Language group	Annotators	Media articles	Manifestos	Total texts	Mean texts per coder	Total sentences	Mean sentences per coder
Bulgarian	5	226	34	260	52	6919	1384
German	10	221	40	261	26	9183	918
Greek	5	298	30	328	66	7349	1470
English	10	373	35	408	41	10,305	1031
French	6	185	34	219	37	4650	775
Hebrew	9	214	36	250	28	7331	815
Italian	8	245	31	276	35	6379	797
Dutch	4	293	30	323	81	10,982	2746
Turkish	9	299	24	323	36	11,133	1237
Total	66	2354	294	2648	40	74,231	1125

#### Annotation validation and curation

To assess agreement between annotators, inter-annotator agreement (IAA) was estimated using Krippendorff’s α for all annotator pairs within each language group at both the level of the ten basic values (Schwartz, 1992) and the 19 refined values (Schwartz et al., 2012).^15^ Agreement was calculated for annotated text spans with at least 50% word overlap (see Table 4 for examples). This threshold was selected to balance comparability and flexibility, allowing for minor boundary differences in span selection while still requiring substantial overlap in annotated content. Empirically, it approximately doubled the number of comparable annotation pairs relative to exact matching, with only a negligible effect on IAA estimates (Δ ≈ 0.02).

**Table 4:** Example of IAA cases subject to span overlap

status: AGREE	
text_1: The federal waiver will allow states to avoid requiring applicants to have greater knowledge on the maintenance of a bus engine components	label_1: Conformity-rules
text_2: avoid requiring applicants to have greater knowledge on the maintenance of a bus engine components	label_2: Conformity-rules
status: DISAGREE	
text_1: we will get additional, qualified drivers behind the wheel to get kids to school safety	label_1: Universalism-concern
text_2: By allowing states to focus on the testing requirements that are critical to safety, we will get additional, qualified drivers behind the wheel to get kids to school safety	label_2: Security-societal

As shown in Table 5, across all annotations, α = 0.546 for the basic values and α = 0.486 for the refined values. These values fall below the commonly cited threshold of α = 0.667 (Piskorski et al., 2023), indicating moderate agreement. Variation in IAA was observed across language groups (i.e., basic values: α = 0.367 to 0.696; refined values α = 0.340 to 0.680), with the Greek and French groups exceeding this threshold (α = 0.696 and α = 0.685, respectively).^16^

**Table 5:** Krippendorff’s alpha’s for annotations by language group

	BG	DE	EL	EN	FR	HE	IT	NL	TR	All
α basic values	0.495	0.367	0.696	0.409	0.685	0.557	0.610	0.411	0.463	0.546
α refined values	0.473	0.340	0.680	0.371	0.656	0.533	0.609	0.380	0.408	0.486

BG = Bulgarian, DE = German, EL = Greek, EN = English, FR = French, HE = Hebrew, IT = Italian, NL = Dutch, TR = Turkish

Importantly, these levels of agreement reflect the inherently interpretive nature of value annotation. In line with recent perspectivist approaches to subjective annotation (Cabitza et al., 2023), the annotation process was designed to focus on consistency but not at all costs. The process targeted a “ground truth” with calibration procedures, including shared training, iterative guideline development, and regular cross-language discussions, aimed to align annotators on theoretical principles but still allowing some limited degree of flexibility in ambiguous or context-dependent cases.

Disagreements were subsequently resolved during a structured curation phase, in which expert curators adjudicated annotations with reference to established guidelines and precedents. This process was intended to ensure theoretical coherence and cross-lingual comparability, rather than impose a single perspective. Accordingly, the IAA scores reported here should be interpreted as reflecting pre-curation variation in annotations, not the final level of consistency in the curated dataset. The curation procedure is described in the following subsection.

##### Curation

To ensure the integrity and consistency of the dataset, all annotations were subject to a structured curation process within each language group. Where annotators independently selected the same text span and assigned the same value and attainment label, these annotations were automatically accepted, following the principle that independent agreement provides evidence of annotation reliability (Artstein & Poesio, 2008). Similarly, text segments that were not annotated by multiple annotators were treated as consensual instances of no value expression.

Curation was required in cases of disagreement, including (1) partial or non-overlapping span selections within the same sentence, and/or (2) differing value or attainment labels assigned to the same or overlapping text spans. These cases were adjudicated by trained curators (domain experts) using a structured procedure. Rather than relying on ad hoc judgment, curators resolved disagreements through systematic reference to the annotation guidelines, documented precedents from prior adjudications, and decisions established in regular cross-language calibration meetings. When necessary, ambiguous cases were escalated to discussion at the project team meetings to ensure consistent application of the coding framework. Curators could either select the most appropriate value label for a span, refine span boundaries, or discard the annotation if the value expression was not sufficiently supported (cf. Bayerl & Paul, 2011; Talat & Hovy, 2016).

This process was designed to prioritize theoretical coherence and consistency across annotators and languages, while remaining grounded in explicit guidelines and shared decision rules. In line with perspectivist approaches to subjective annotation (Cabitza et al., 2023), the goal of curation was to reconcile interpretations within a common theoretical framework. As such, curation can be understood as a form of expert-guided alignment rather than unilateral decision-making. The resulting dataset reflects annotations that have been both independently generated and systematically reviewed, reducing noise while preserving meaningful variation across contexts.

In addition to within-language curation, we implemented a cross-lingual quality assurance step to assess the consistency of annotations across languages. Following Stefanovitch et al. (2023), we applied a holistic IAA approach to clusters of semantically similar text spans. First, English translations of annotated spans were compared, and clusters with at least 50% word overlap were identified. Second, clusters with differing value labels were flagged for manual review. Third, language leads from the relevant groups jointly evaluated these cases to assess the appropriateness of annotations within their linguistic and cultural context (see Table 6 for an example). This procedure enabled the identification of systematic discrepancies in value interpretation across languages and supported improved cross-lingual alignment. While this step did not alter the number of annotations, it strengthened the overall coherence and comparability of the dataset.

**Table 6:** Example of a multilingual cluster annotated in Dutch, German, and Greek

Value	Language	Annotated text (English translation)	Annotated text (Original)
Power-dominance	Greek	Recep Tayyip Erdogan’s proximity to Vladimir Putin is part of his game	: Η εγγύτητα του Ερντογάν με τον Πούτιν είναι μέρος του παιχνιδιού του «Η εγγύτητα του Ρετζέπ Ταγίπ Ερντογάν με τον Βλαντιμίρ Πούτιν είναι μέρος του παιχνιδιού του» σημειώνει σε άρθρο του στη γαλλική LE MONDE ο Marc Pierini, πρώην Πρέσβης
Universalism-tolerance	German	Desire to bring together Russian leader Vladimir Putin and Ukrainian President Volodymyr Zelenskyi	auf ein Ende des Krieges erforscht. Die Türkei äußerte zuletzt erneut den Wunsch, den russischen Machthaber Wladimir Putin und den ukrainischen Präsidenten Wolodymyr Selenskyj zusammenbringen zu wollen. Der Berater von Selenskyj und zugleich ukrainischer Chefunterhändler Mykh
Conformity-interpersonal	Greek	Ukraine’s leader Voldimir Zelensky and British Prime Minister Boris Johnson have signed a free trade agreement and strategic partnership between the countries	ερα επίπεδα εκπροσώπων των δύο χωρών δεν σπανίζουν. Τον Οκτώβριο του 2020, ο ηγέτης της Ουκρανίας Βόλντυμιρ Ζελένσκι και ο Βρετανός πρωθυπουργός Μπόρις Τζόνσον υπέγραψαν συμφωνία ελεύθερου εμπορίου και στρατηγική εταιρική σχέση μεταξύ των χωρών. Παρά τις προσπάθειες απόκρυψης της επίσημης συνάντησης, έγινε ευρέως γνωσ
Conformity-interpersonal	Greek	U.S. Undersecretary of State Francis Fanon sends a message to Türkiye to de-escalate tensions	κοκυπρίων, θα απαντήσουμε με ανάλογα βήματα σε κάθε αρνητικό βήμα της ΕΕ». Μήνυμα στην Τουρκία να αποκλιμακώσει την ένταση, έστειλε από την Λευκωσία, ο υφυπουργός εξωτερικών των Ηνωμένων Πολιτειών, Φράνσις Φάνον. «Καλούμε όλα τα μέρη να μην προβαίνουν σε προκλητικές ενέργειες που μπορο
Power-dominance	German	In addition to Gabriel, former CDU politician Hildegard Müller will be in the race	Vize-Kanzler sei der Wunschkandidat der Autokonzerne und der Zulieferer. Neben Gabriel soll die frühere CDU-Politikerin Hildegard Müller im Rennen sein, wie die Frankfurter Allgemeine Sonntagszeitung am Wochenende berichtete
Face	German	He finished his short lecture with the exclamation “Slawa Ukrajini”, “Glory of Ukraine”, which was popularized by Ukrainian President Volodymyr Zelenskyi	Kompromisse auf Kosten der Ukraine zeugten von einer politischen Naivität. Seinen Kurzvortrag beendete er mit dem Ausruf „Slawa Ukrajini“, „Ruhm der Ukraine“, der vom ukrainischen Präsidenten Wolodymyr Selenskyj populär gemacht wurde. Ob Russland Atomwaffen einsetzen werde, wollte Dengel anschließend von
Tradition	Dutch	my uncle, Jef Tavernier, at the cradle of Agalev and my cousin, Maarten Tavernier, was the first successor to the Flemish Green list in West Flanders	jn moeders kant draagt een sterke Vlaamse stempel. Langs vaders kant stond mijn oom, Jef Tavernier, aan de wieg van Agalev en mijn neef, Maarten Tavernier, was de voorbije verkiezingen eerste opvolger op de Vlaamse lijst van Groen in West-Vlaanderen.’ Zelf voelde Tavernier zich al snel thuis bij N-VA. Na haar studies maakte

### The value annotations dataset

This section describes the complete, curated dataset, released as *Touché24-ValueEval* as part of the ValueEval’24^17^ competition (Kiesel et al., 2024). The dataset provides a large-scale, multilingual resource for analyzing value expression in political text. It is presented in tabular format, with each row corresponding to a sentence. Texts were automatically split into sentences using Trankit (Nguyen et al., 2021; v1.1.1). When annotations span multiple sentences, the corresponding value label was assigned to each sentence. The dataset comprises 2648 texts and 74,231 sentences across nine languages, with aligned English translations^18^ provided in the same format to facilitate cross-lingual analysis.

**Fig. 3:**
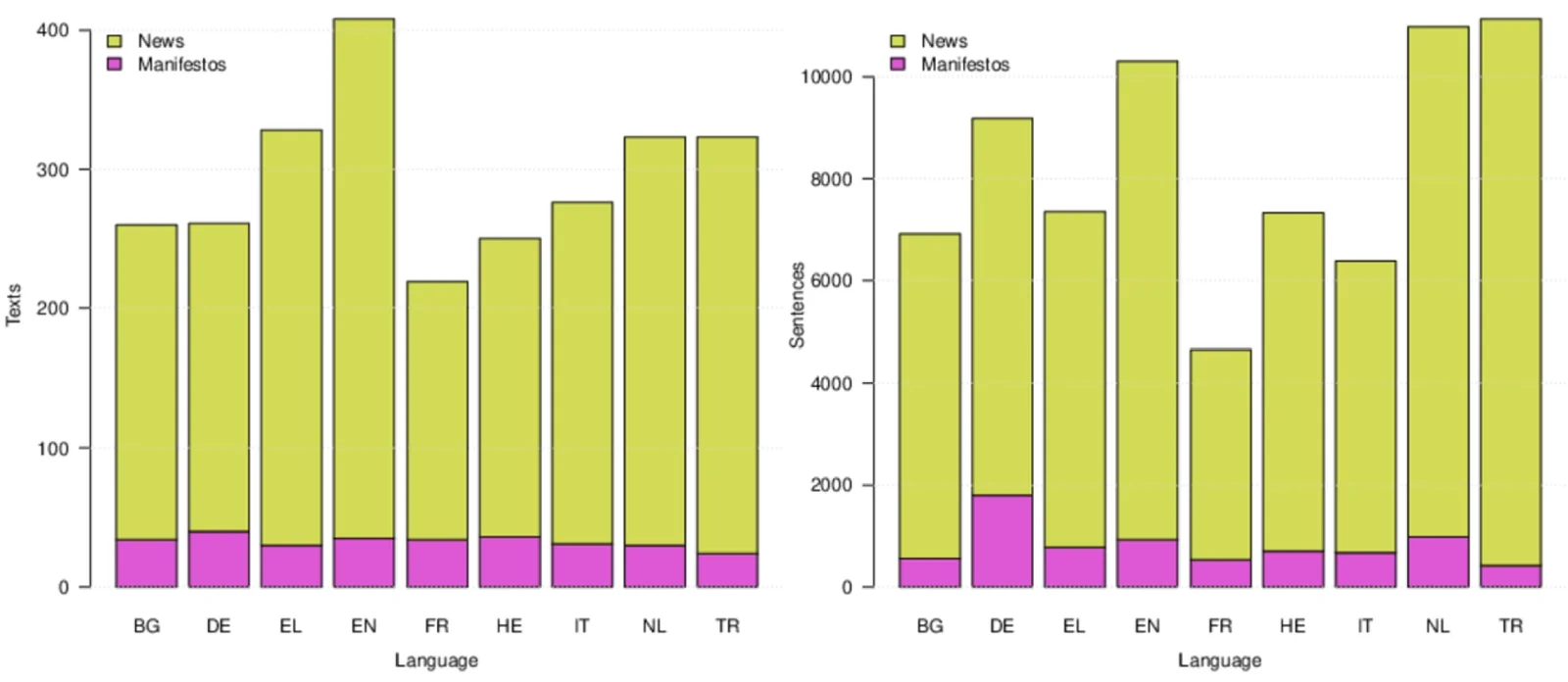
Number of Texts (***left***) and Sentences (***right***) per Language in the dataset. *Note*. News articles = yellow, political manifestos = pink. *N* = 2648 texts. *N* = 74,231 sentences. BG = Bulgarian, DE = German, EL = Greek, EN = English, FR = French, HE = Hebrew, IT = Italian, NL = Dutch, TR = Turkish

**Fig. 4:**
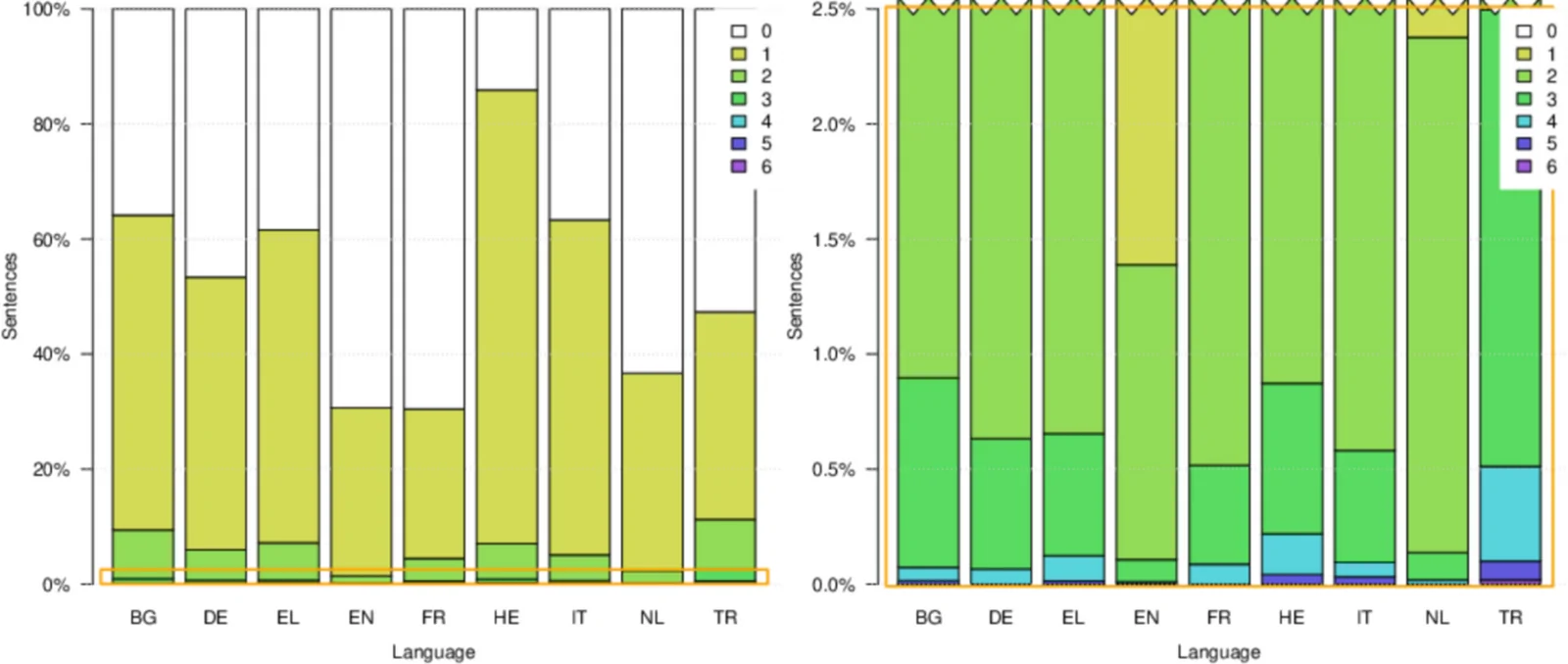
Percentage of sentences with the number of values annotated. *Note*. Share of refined value counts per sentence per language in the dataset. *Left*: the absolute share, *right*: the percentage area between 0 and 2.5%. BG = Bulgarian, DE = German, EL = Greek, EN = English, FR = French, HE = Hebrew, IT = Italian, NL = Dutch, TR = Turkish, 0 meaning no value annotation, 6 meaning 6 value annotations per sentence

As shown in Figs. 3 and 4, the distribution of texts and sentences is broadly comparable across language groups, with French contributing the fewest annotations and English the most. At the sentence level, most instances were annotated with either a single dominant value or no value. Although multiple values may be expressed within a sentence, the annotation protocol prioritized the identification of the dominant value to enhance consistency, reduce ambiguity, and support downstream computational modeling. Curators were instructed to assign multiple values only where distinctions were unclear, resulting in a concise yet expressive representation of value content (see Fig. 4; Supplementary Material, Annex 4 & 5).

**Fig. 5:**
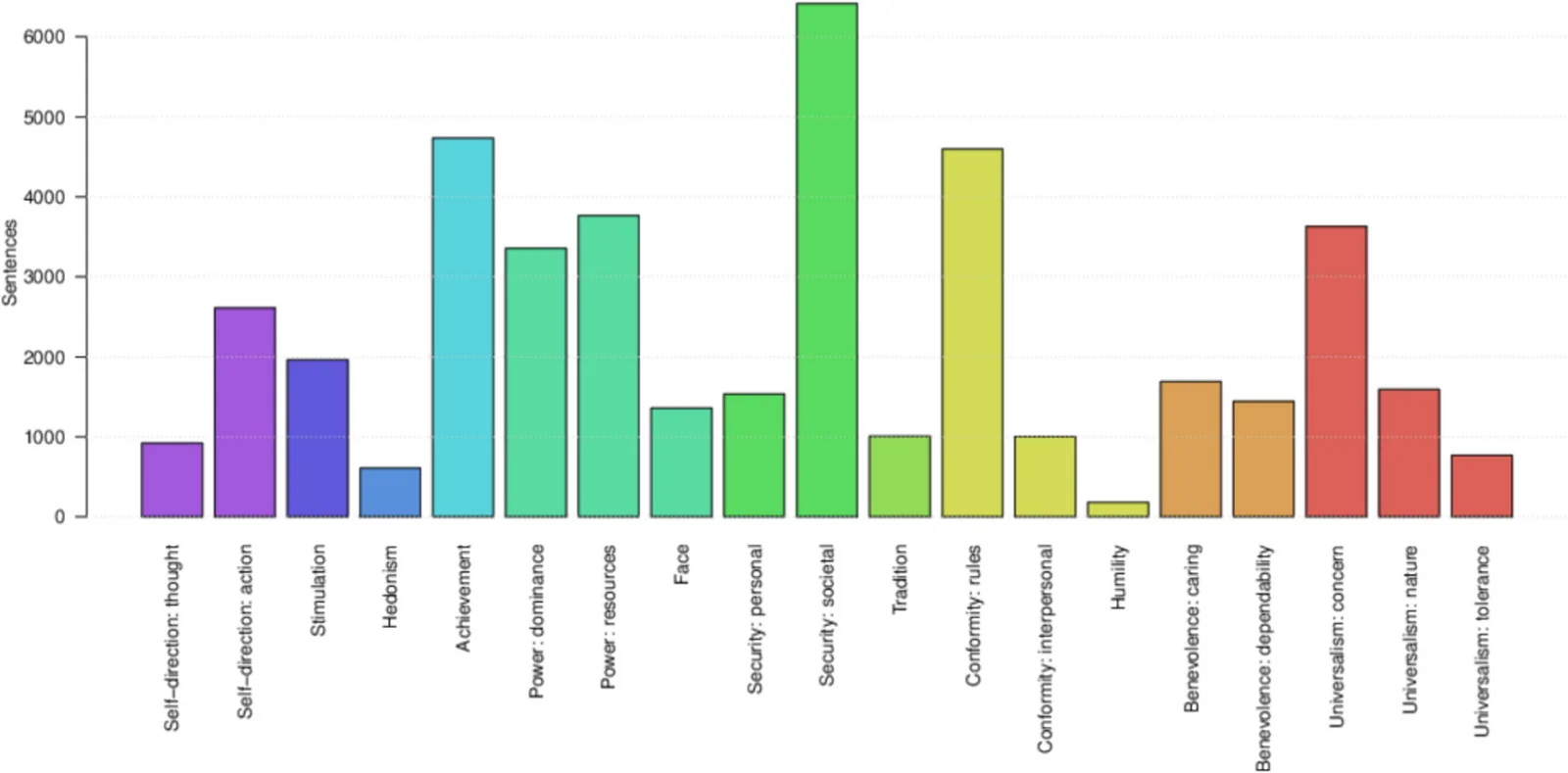
Overall count of refined value annotations

Figure 5 presents the distribution of the 19 refined values across the dataset, showing substantial variation both between and within higher-order value domains. Security-societal emerges as the most frequently annotated value, followed by achievement and conformity-rules, whereas hedonism, humility, and tradition are least represented. This pattern reflects the corpus's political focus, which emphasizes societal and institutional concerns. At the same time, the dataset captures fine-grained distinctions within value domains, enabling analysis at both the basic and refined value levels.

**Fig. 6:**
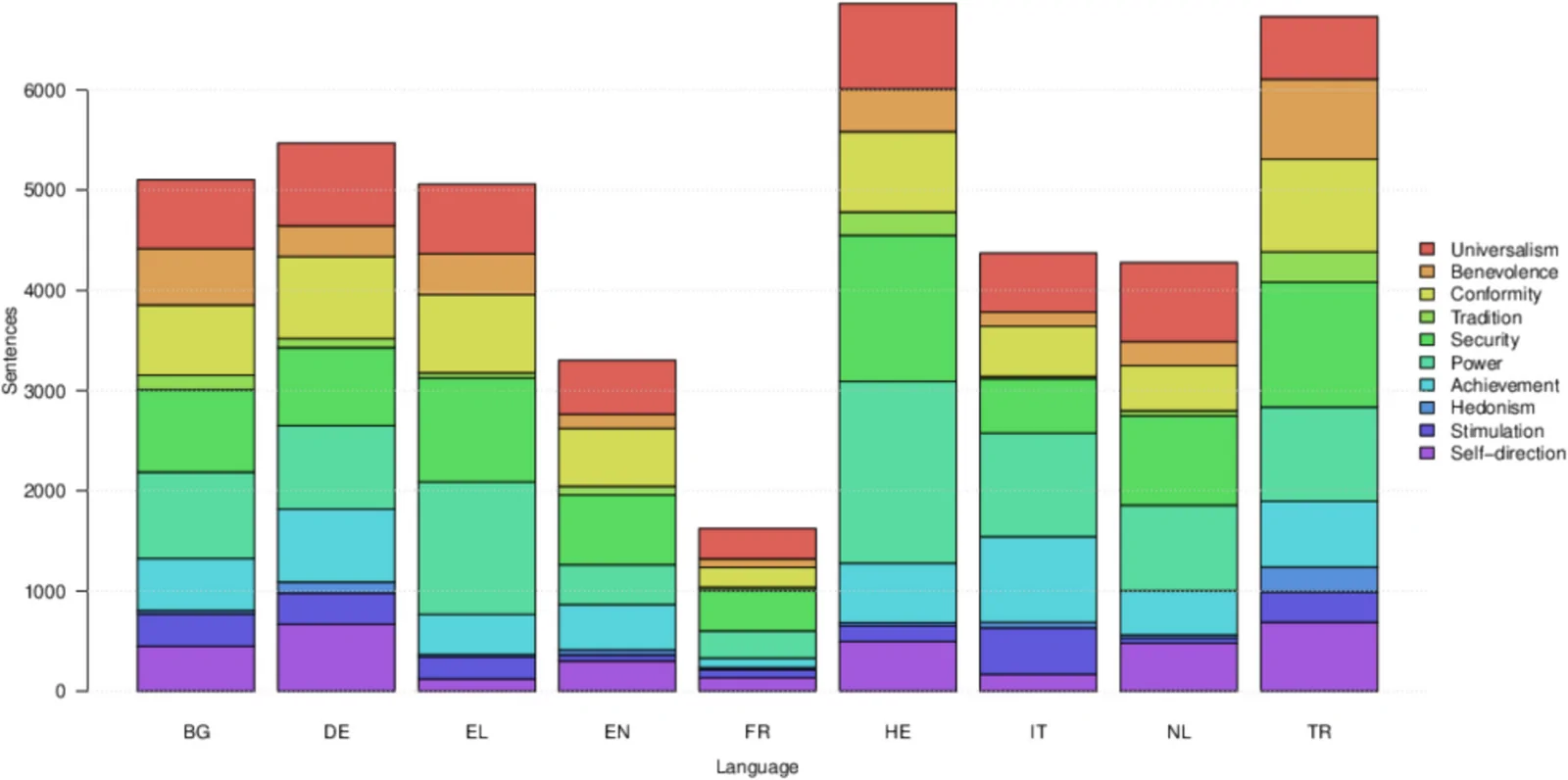
Frequency of basic value annotations by language group. *Note*. BG = Bulgarian, DE = German, EL = Greek, EN = English, FR = French, HE = Hebrew, IT = Italian, NL = Dutch, TR = Turkish

Figure 6 presents the distribution of basic value annotations across language groups. While overall annotation frequencies vary between languages (e.g., higher in Hebrew than in French), there is also variation in the prevalence of specific values. For example, self-direction values are more frequently annotated in Turkish than in Greek. Despite these differences, a consistent pattern emerges across languages: security, power, universalism, and conformity are the most frequently annotated basic values, whereas tradition and hedonism are the least represented. This pattern suggests that political discourse across contexts is structured around core societal concerns, such as security, social order, and collective welfare, rather than values associated with personal gratification or convention.

**Fig. 7:**
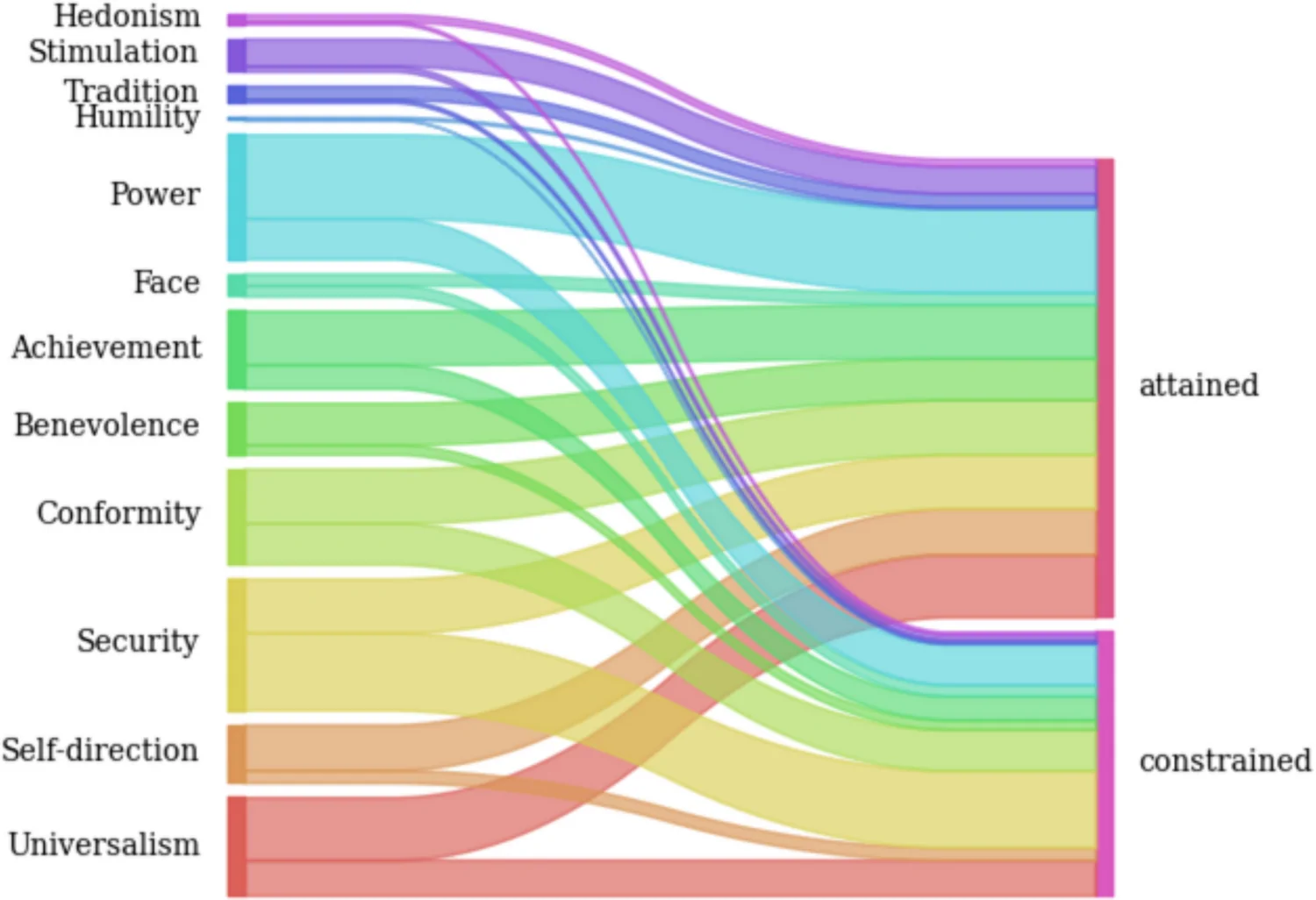
Level of attainment for basic values. *Note*. Each value falls into “(partially) attained”, “(partially) constrained” and “Don’t know/undecided”. The latter category was assigned as 0.5 weight to both attainment levels

Annotations also include an attainment label indicating whether values are expressed as (partially) attained, (partially) constrained, or undecided. In Fig. 7, undecided cases are evenly split between attained and constrained (0.5 each).^19^ All basic values were annotated in both attained and constrained forms, indicating that this distinction is meaningful and analytically useful. Overall, values were more frequently annotated as attained than constrained. This pattern is consistent with the conceptualization of values as desirable end-states (Rokeach, 1973; Schwartz, 1992) and with the annotation guidelines, which prioritize identifying attained values in cases of ambiguity. An exception is observed for security, which was more frequently annotated as constrained, potentially reflecting the prominence of threat framing in political discourse where values are invoked through perceived risks and violations (e.g., Scheller, 2019).

**Fig. 8:**
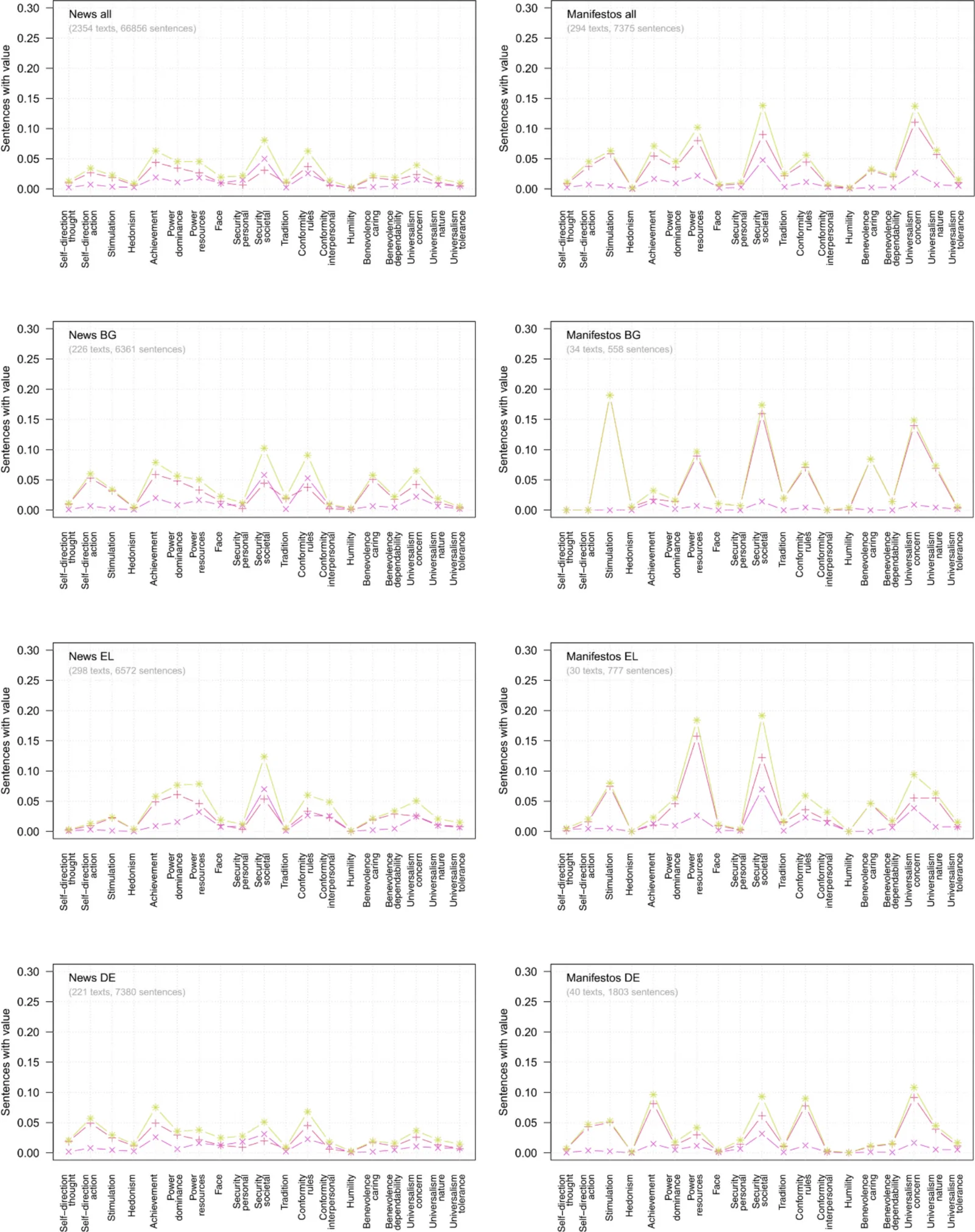

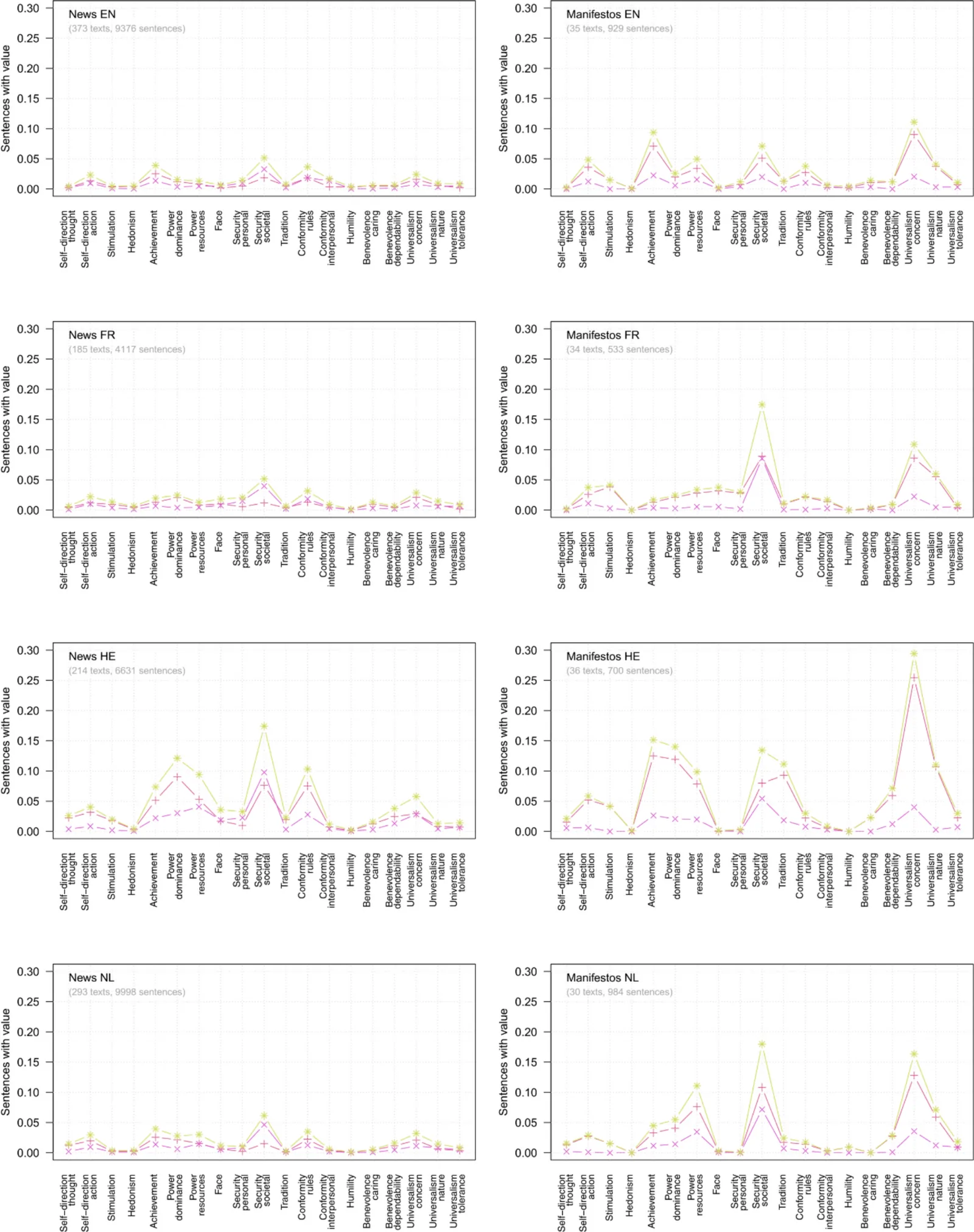

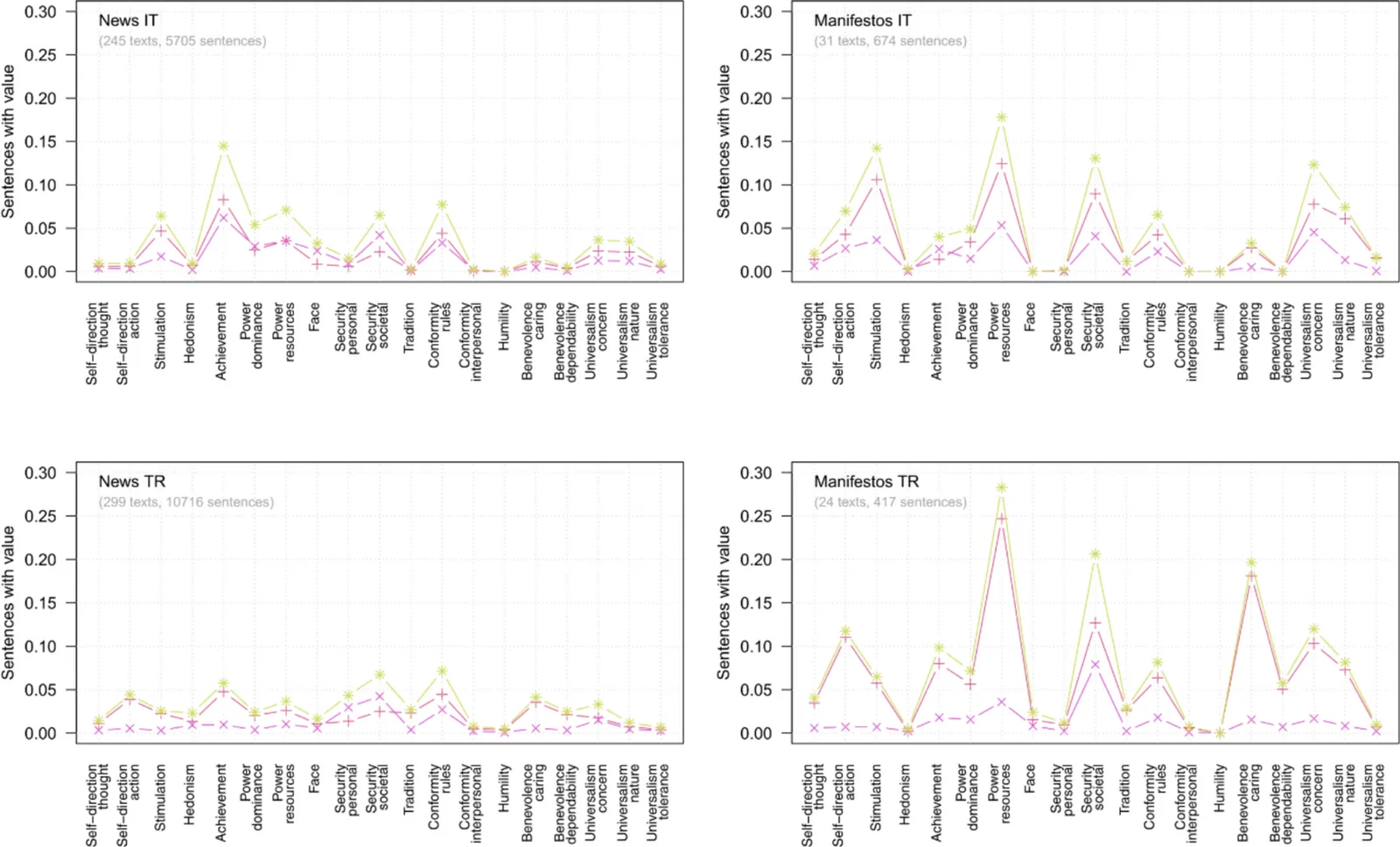
Proportion of sentences expressing each value in news articles (*left*) and manifestos (*right*), by language and attainment level (attained = +, constrained = ⨉, combined = ✴).* Note*. BG = Bulgarian, DE = German, EL = Greek, EN = English, FR = French, HE = Hebrew, IT = Italian, NL = Dutch, TR = Turkish

**Fig. 9:**
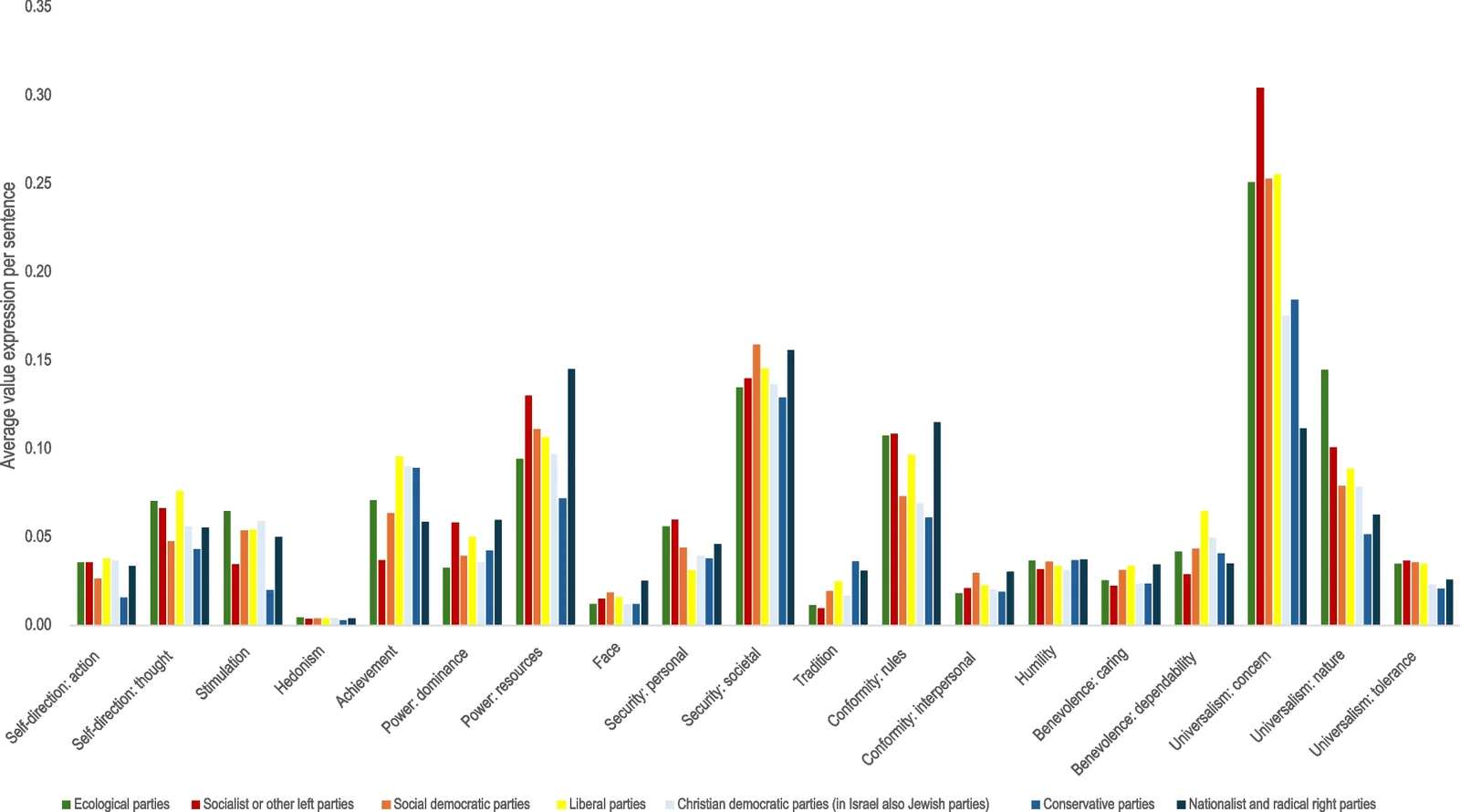
Average value expression per sentence in political manifestos. *Note*. Per-sentence value expression in 2020 political manifestos from France, Germany, Greece, the Republic of Ireland, Italy, the Netherlands, and the United Kingdom, estimated using the winning algorithm trained on this dataset (i.e., Legkas et al., 2024). Values are averaged across countries, parties within each political family (as classified by the Manifesto Project; Lehmann et al., 2025), and sentences. Agrarian, special-issue parties, and ethnic/regional parties are excluded for clarity

Figures 8, 9 provides a more detailed comparison by language, text type, refined value, and attainment level. Across languages, value annotations are generally more frequent in manifestos than in news articles, although this pattern varies by value type. In particular, refined values in the self-transcendence dimension (from humility to universalism) are substantially more prevalent in manifestos, suggesting systematic differences in how values are expressed across political text genres. This suggests that political actors strategically emphasize values related to collective welfare and moral ideals in programmatic texts, whereas news reporting may foreground a broader or more neutral range of value expressions. Together, these patterns illustrate how value expression is shaped not only by cultural context but also by the communicative function of political texts.

#### Assessing external validity

As a first step in assessing external validity, we examine the co-occurrence of values in the annotated dataset and evaluate whether these patterns align with the theoretical structure of the values circumplex (Schwartz, 1992; see Table 7). Absolute co-occurrence frequencies were relatively low, as the annotation guidelines prioritized assigning a single dominant value per sentence. We therefore report conditional co-occurrence ratios indicating how much more likely two values are to co-occur relative to their baseline frequency. For example, a value of 2.41 for hedonism-tradition indicates that, conditional on hedonism being present, tradition is 2.41 times more likely to co-occur than expected by chance. In contrast, a value of 0.21 for power-hedonism indicates that these values co-occur substantially less frequently than expected based on their position in the Schwartz (1992) circumplex. Annex 7 also presents word clouds for the most expressed words in each value span to provide some understanding of which words are seen as representing values most topically.

**Table 7:** Conditional probability factor of co-occurrence between values at the sentence level

	Self-direction	Stimulation	Hedonism	Achievement	Power	Face	Security	Tradition	Conformity	Humility	Benevolence	Universalism
Self-direction	-	0.76	0.73	0.48	0.54	0.62	0.52	0.73	0.64		0.80	0.58
Stimulation	0.76	-	1.42	0.88	0.38	0.44	0.39	0.45	0.27		0.79	0.71
Hedonism	0.73	1.42	-	0.74	0.21		0.74	2.41	0.39		0.66	0.47
Achievement	0.48	0.88	0.74	-	0.47	0.74	0.32	0.57	0.26	0.87	0.52	0.43
Power	0.54	0.38	0.21	0.47	-	0.53	0.45	0.50	0.43		0.55	0.52
Face	0.62	0.44		0.74	0.53	-	0.37		0.60		0.65	0.51
Security	0.52	0.39	0.74	0.32	0.45	0.37	-	0.40	0.49	0.78	0.51	0.45
Tradition	0.73	0.45	2.41	0.57	0.50		0.40	-	0.33		1.18	0.81
Conformity	0.64	0.27	0.39	0.26	0.43	0.60	0.49	0.33	-		0.54	0.60
Humility				0.87			0.78			-	1.32	
Benevolence	0.80	0.79	0.66	0.52	0.55	0.65	0.51	1.18	0.54	1.32	-	0.68
Universalism	0.58	0.71	0.47	0.43	0.52	0.51	0.45	0.81	0.60		0.68	-

Values indicate how much more a value (column) occurs given another value (row), relative to its overall frequency. Cases with fewer than ten sentences per value are excluded

Overall, several co-occurrence patterns are consistent with the circumplex structure of values, including higher conditional co-occurrence among adjacent or compatible values (e.g., hedonism-stimulation, tradition-benevolence, humility-benevolence) and lower conditional co-occurrence among opposing values (e.g., universalism-achievement, conformity-stimulation, conformity-achievement). At the same time, some unexpected associations emerge, notably, the elevated co-occurrence of tradition and hedonism appears to reflect specific contextual patterns in the data, such as references to traditional festivities, national celebrations, and culturally valued practices. These findings suggest that while value co-occurrence broadly reflects theoretical expectations, contextual instantiations of values can generate deviations from the idealized circumplex structure.

Next, we investigate patterns of value expression across political parties. Figure 9 presents the average value expression per sentence in political manifestos in 2020, grouped by political ideology across all countries in the dataset. Overall, the observed patterns are broadly consistent with theoretical expectations. Liberal parties score highest on self-direction (action and thought) and achievement, while nationalist and radical right-wing parties score highest on power (dominance and resources), face, conformity (rules and interpersonal), humility and benevolence-caring. Social democratic and other left-wing parties are characterized by higher levels of universalism (concern and tolerance) and security-personal, whereas ecological parties emphasize universalism-nature and, to a lesser extent, hedonism. Conservative parties exhibit the strongest association with tradition.

These patterns are broadly in line with the higher-order value dimensions proposed by Schwartz (1992), particularly the conflicts between self-transcendence and self-enhancement, and openness to change and conservation. For example, left-leaning and ecological parties appear to emphasize self-transcendence values more strongly, whereas right-leaning parties tend to emphasize conservation and self-enhancement values more strongly. More generally, parties with more clearly defined ideological positions appear to exhibit more distinct value profiles, suggesting that value expression in manifestos may reflect the articulation of ideological priorities. Taken together, these patterns provide tentative support for the external validity of the dataset, indicating that aggregated value annotations correspond meaningfully to established ideological distinctions across political families.

Taken together, the value annotations dataset combines scale, theoretical grounding, and multilingual coverage to provide a novel resource for the study of value expression in political text. By linking values to contextualized text spans across diverse sources, it enables systematic analysis of value dynamics in political communication. In addition to its descriptive utility, the dataset serves as a benchmark for the development and evaluation of computational models of value detection, supporting more transparent, theory-grounded, and cross-lingually comparable analyses of normative content. These features provide a foundation for future work on the measurement, interpretation, and modeling of values in text, which we consider in the following section.

## Discussion

We present a new annotated dataset of value expression in political text (news articles and political manifestos), comprising 74,231 sentences across nine languages. Grounded in Schwartz et al.’s (2012) refined values theory, the dataset provides a structured multilingual resource for analyzing how values are expressed in political discourse. This responds to evidence that citizens respond to political cues through motivated and identity-based reasoning processes (Leeper & Slothuus, 2014; Van Bavel & Pereira, 2018) and that political preferences are structured by underlying values (e.g., Barnea & Schwartz, 1998; Caprara et al., 2006). By combining theory-driven annotation guidelines with iterative training, calibration, and expert curation, this work contributes to efforts in computational social science to operationalize values in naturally occurring text.

A key contribution is the development of a scalable annotation framework that balances theoretical precision with practical feasibility. The use of refined values enables fine-grained distinctions, while the addition of attainment labels captures whether values are framed as attained or constrained. This extends prior work on value instantiations (Hanel et al., 2018a, 2018b; Maio et al., 2009; Ponizovskiy et al., 2019) and aligns with evidence that value framing can shape attitudes and evaluations (Maio et al., 2009). At the same time, the framework presented in this paper explicitly acknowledges the interpretive nature of value annotation. Value expressions in text are often polysemous, and multiple values may plausibly be inferred from a single span of text. To address this, annotators were trained to prioritize the evaluative meaning most directly expressed in the text, allow multiple value annotations when clearly present, and use a dominant value to enhance consistency in ambiguous cases.

Several methodological considerations follow. First, moderate inter-annotator agreement reflects the inherent ambiguity and context-dependence of identifying value expression, particularly across languages and political contexts. Disagreement should therefore not be viewed solely as measurement error, but as indicative of interpretive complexity.

Our annotation design follows established practice in large-scale NLP dataset construction (e.g., Hovy et al., 2006; Pradhan et al., 2013), whereby expert annotators code subsets of data and reliability is achieved through shared guidelines, training, and structured overlap across coders rather than requiring many annotators per text. This approach is exemplified by resources such as OntoNotes, which combine expert annotation, calibration, and adjudication to ensure consistency at scale. In the value annotations dataset, most texts were annotated by two experts, although a small proportion (12%) were annotated by a single expert, which we acknowledge as a limitation. Disagreements were resolved through expert curation, with curators systematically applying established guidelines and precedents from ongoing calibration discussions to ensure consistency and theoretical alignment.

While this approach prioritizes cross-lingual comparability and coherence, it may also introduce curator-driven bias by favoring consistent interpretations over annotator variability. Accordingly, the resulting annotations should be understood as theory-guided, context-sensitive approximations rather than definitive classifications, consistent with prior work showing that value interpretation can vary across cultural contexts (Schwartz & Sagiv, 1995).

Second, variation in annotation rates and value distributions across language groups highlights the role of contextual and linguistic factors. Differences in media coverage, discourse conventions, and annotator sensitivity may all influence the likelihood that values are identified in text. Given that news media play a key role in shaping and mediating political information (McCombs & Valenzuela, 2020; van der Meer et al., 2023), such variation likely reflects both substantive differences in discourse and methodological constraints.

Third, the corpus was intentionally designed to capture value expression in political and policy-relevant text. Topic categories were selected to ensure comparability across languages in domains where values are explicitly debated (e.g., immigration, economy, environment, health). While this supports cross-linguistic consistency, it does not capture the full range of value expression across all domains. Values expressed in areas such as the arts, sport, or interpersonal relationships are therefore underrepresented, and variation in topic distributions across languages reflects differences in media coverage and sampling constraints rather than theoretical imbalance. The dataset should thus be interpreted as a resource for analyzing value expression in political communication rather than as a comprehensive representation of all value-relevant discourse.

A further consideration concerns the relationship between annotated values and actors’ explicit claims. In politically contested domains, statements may be framed in terms of particular values while also reflecting broader value structures that can be interpreted differently within a theoretical framework. The present approach focuses on coding the evaluative content expressed in text, rather than inferring underlying intentions. As such, annotations represent structured, theory-informed interpretations of value expression, which may not always align with actors’ self-descriptions. This distinction aligns with broader concerns about variability and potential bias in expert judgment across complex evaluative domains (Cabitza et al., 2023).

These considerations have implications for the use of the dataset. Value annotations should be interpreted as probabilistic and context-dependent indicators rather than exhaustive representations of meaning. To support robust applications, we recommend several quality checks, including examining value distributions and co-occurrence patterns for theoretical plausibility, assessing consistency across languages and subsets, and comparing results with alternative approaches (e.g., dictionary-based measures, see Ponizovskiy et al., 2020). The quality checks reported in this paper yielded theoretically consistent patterns, and supplemental analysis of annotators’ personal value priorities showed weak and unsystematic associations with annotation outcomes (see Supplementary Materials, Annex 6). However, researchers are encouraged to triangulate findings with complementary approaches (e.g., Stecker & Hopp, 2026), particularly in politically sensitive domains.

To further assess the validity and robustness of these annotations, the dataset also enables several concrete validation tests. A key prediction is that value expression in text should reproduce the circumplex structure of values, such that opposing values (e.g., self-transcendence vs. self-enhancement; openness vs. conservation) exhibit negative associations, while adjacent values show positive associations. Convergent validity can be assessed by comparing aggregate value profiles derived from text with external benchmarks, such as survey-based measures of value priorities (e.g., values from the European Social Survey or World Values Survey) or macro-level indicators (e.g., gross domestic product). In addition, robustness can be evaluated by comparing expert annotations with lay judgments and alternative approaches (e.g., dictionary-based methods, large language models), as well as by testing whether models trained on the dataset generalize to out-of-sample corpora. Together, these approaches provide testable pathways for assessing the structural validity, convergence, and generalizability of annotated value expression.

Despite the considerations for use of the dataset, it offers a unique combination of theoretical grounding, multilingual coverage, and contextual richness. It enables systematic analysis of value expression in political text across countries and text types, including both news and manifestos, which may differ in political expressiveness (Blokker et al., 2020). The dataset also provides a benchmark resource for developing and evaluating computational models of value detection, including recent advances in multilingual modeling (Kiesel et al., 2024; Legkas et al., 2024), and can contribute to ongoing efforts to align AI systems with societal values (Yao et al., 2023).

Future research can extend this work in several directions. Expanding the corpus into additional domains would enable a more comprehensive assessment of value expression across contexts. Further work is also needed to examine cross-cultural differences in annotation practices and to refine methods for capturing value co-occurrence and ambiguity. Finally, integrating text-based measures with survey and behavioral data offers a promising avenue for advancing the study of values and their role in shaping political and social outcomes.

## Supplementary Information

Below is the link to the electronic supplementary material.

Supplementary file1 (DOCX 1805 KB)

## Data Availability

The data is available here : https://zenodo.org/records/13283288. Instructions and materials for developing the data are published under Reitis-Münstermann et al. (2024). The data were collected solely with the authors' consent, who hereby give consent to its publication.

## Footnotes

1 Crowd workers undertook the annotation task by choosing value expressions from a list of 54 value statements developed from survey items measuring the 19 refined values (such as “Have life accepted as is” for the value “humility”) and an additional value "universalism objectivity".

2 The research team considered alternative ways of coding the evaluative quality of value expression (e.g., whether the value was framed positively or negatively). However, pilot testing indicated that the more informative distinction concerned the extent to which a value was expressed as being attained or constrained. This approach is consistent with Schwartz’s theory, which conceptualizes values as desirable end states, and with related work in MFT that distinguishes between “virtues” and “vices” as opposing expressions of the same underlying moral dimension (Hopp et al., 2021).

3 Fifteen curators also contributed as annotators within their respective language groups; in these cases, their annotations were independently curated by another curator to ensure consistency and avoid self-review.

4 https://data.jrc.ec.europa.eu/collection/emm

5 For all language groups the political manifestos and news media sources came from the country of origin (i.e., Bulgarian political parties and Bulgarian news media for the Bulgarian language group). The exception was the English language group where the majority of news media texts were drawn from sources in the Republic of Ireland and political manifestos were drawn from the United Kingdom and the USA.

6 Mainstream sources are based on a manually curated list of 465 EU27 online sources monitored by the EMM and represent the most popular open news websites in each EU Member State in terms of visitors. The number of outlets per country was weighted based on the countries’ population.

7 These sources monitored in the EMM have been identified by independent fact-checkers and other independent experts as frequently spreading extreme views and mis- or disinformation.

8 EMM includes keyword-based labelling of articles in their processing chain for the selected sources.

9 The EMM database is compiled via automated web scraping of thousands of news websites and blogs across more than 50 languages worldwide (Steinberger et al., 2013). As a result, the data are inherently noisy, requiring substantial manual cleaning, with variability in data quality across languages.

10 Due to successive manual cleaning and selection steps, the initial randomization of topics and articles within topics may be partially attenuated.

11 For the French language group, manifestos from the presidential election were used.

12 Flashcards were provided in English (238 items across four levels), French (63 items, level 2), and Italian (198 items, levels 2, 3, and 4).

13 State of the European Union Speech 2022 by Ursula von der Leyen: https://ec.europa.eu/commission/presscorner/detail/en/speech_22_5493

14 https://inception-project.github.io/

15 Annotations for the ten basic values were derived by aggregating counts of annotations for the corresponding refined values (e.g., annotation counts for security-societal and -personal were summed to create a count for the basic security values). In this procedure, humility was included in conformity, and face within power, consistent with the structure of values.

16 Although IAA estimates are reported based on the full set of completed annotations, α values remained relatively stable within each language group throughout the annotation campaign. We also tested for Prevalence and Bias Adjusted Kappa (PABAK), but the results are not qualitatively different from Cohen’s kappa.

17 https://valueeval.webis.de

18 Using Google’s Translation AI API (https://cloud.google.com/translate) for Hebrew and the DeepL API (https://www.deepl.com/en/pro-api) for all other languages (DeepL did not support Hebrew at that time).

19 Annex 4 in Supplemental Materials shows the exact numbers per value.

## Code availability

The code is available here: https://osf.io/hbdrs/?view_only=7a69eb06694648399c812c2e2034cf52.
