# Supplementary material for: ValuesML: A new multilingual dataset for values detection in news and political manifestos
Source: Behav Res Methods. 2026 Aug 20;58(10):276. doi: 10.3758/s13428-026-03092-z (PMC13493494; doi:10.3758/s13428-026-03092-z)
Supplement: Supplementary file 1 [file 13428_2026_3092_MOESM1_ESM.docx]

**Supplementary Materials**

# **Annex 1: Flash Cards**

**1A: Flash Cards Overview Screen**

**1B: Flash Cards Example Case Non-selected**

**1C: Flash Cards Case Selected**

# **Annex 2: Example of the Spreadsheet used for the Manual Selection of Articles with Instructions**

# **Annex 3: Examples of difficult cases**

| **Issue type** | **Issue description** | **Language group** | **Value** | **Attainment** | **Suggestions / ideas** | **Reporter** | **Agreement in LL meeting** | **agreed on** |
| --- | --- | --- | --- | --- | --- | --- | --- | --- |
| Values | >>He must learn not to have the same bad experiences repeatedly.<< | French | Stimulation | (partially) attained | Stimulation # partially attained is not expressed here. |  | Hedonism constrained also possible, as the person does not have fun Missing context, if person got hurt (Security constrained) if bored: Stimulation as it is about a new experience | 26/06/2023 |
| values | While effective COVID-19 vaccines were developed rapidly, all steps have been taken to ensure their safety and effectiveness | English | Achievement | (partially) attained | security is also attained |  | Achievement; as developed rapidly and safe vaccine second value: Security | 26/06/2023 |
| Values | We support our allies *the best we can* | English | Benevolence–dependability | (partially) attained | benevolence caring is also attained |  | Main value: Benevolence Dependability Also present B. Caring | 26/06/2023 |
| Values | In totalitarian states like this, censorship, and restrictions on freedom of expression dominate the press landscape. | English | Self-direction–action | (partially) constrained | could also fit self direction thought (constrained). Not sure if it is a mistake to code it either way. |  | Both values fit, main value SD Action as it is freedom of expression not of thought | 26/06/2023 |
| Values | Trendsetters are people required for social change – they are the ones who can overcome normative pressure more easily, and the ones who take on the largest risks. | English | Self-direction–action | (partially) attained | risk taking is also related to stimulation |  | Both values are present | 26/06/2023 |
| Values | Flashcards: In totalitarian states like this, censorship, and restrictions on freedom of expression dominate the press landscape. | Greek | Self-direction–action |  | Why not SELF-DIRECTION THOUGHT? (censorship, freedom of thought/expression etc.) |  | Both values fit, main value SD Action as it is freedom of expression not of thought | 26/06/2023 |
| Values | Flashcards: At an early age, the children began to provide for their old age | Greek | Power–resources |  | Why not also SELF-DIRECTION ACTION (e.g. being independent, choosing for oneself, etc)? |  | They provide for themselves more present than being independent. | 26/06/2023 |
| Values | Flashcards: If I continue to go out so often on weekends, I will not pass this semester | Greek | Hedonism |  | I continue to go out so often on weekends -> Hedonism. But in conjunction with the rest of the sentence why not ACHIEVEMENT (negative)? |  | first part of sentence hedonism, second part achievement constrained | 26/06/2023 |
| Values | Flashcards: But right now, it’s the threat to food supplies posed by Vladimir Putin’s Black Sea blockade, and resulting shortages, price inflation and instability, that is the big new factor | Greek | Security–societal |  | Why not also POWER-RESOURCES? Power-resources -> control/dominance over people, resources, economic warfare, etc.? |  | The main message of the sentences is the threat of instability, shortages and inflation. That is a threat to security for all.  The reader is also powerless (so Power constrained) is possible but Security as the immediate consequence During curation, you can keep both values | 03/07/2023 |

# **Annex 4: Number of Annotations by Language and Refined Value**

| Annotation | Bulgarian | German | Greek | English | French | Hebrew | Italian | Dutch | Turkish | Total |
| --- | --- | --- | --- | --- | --- | --- | --- | --- | --- | --- |
| Achievement | 556 | 723 | 411 | 455 | 95 | 552 | 838 | 445 | 730 | 4805 |
| Benevolence-caring | 346 | 160 | 181 | 70 | 54 | 106 | 119 | 52 | 580 | 1668 |
| Benevolence-dependability | 143 | 147 | 239 | 73 | 35 | 269 | 28 | 193 | 302 | 1429 |
| Conformity-interpersonal | 53 | 139 | 354 | 165 | 48 | 71 | 16 | 59 | 87 | 992 |
| Conformity-rules | 629 | 665 | 445 | 382 | 145 | 614 | 454 | 367 | 993 | 4694 |
| Face | 151 | 192 | 130 | 62 | 100 | 202 | 173 | 117 | 197 | 1324 |
| Hedonism | 31 | 104 | 25 | 48 | 28 | 27 | 52 | 33 | 265 | 613 |
| Humility | 18 | 21 | 7 | 38 | 6 | 10 |  | 22 | 54 | 500 |
| Power-dominance | 366 | 292 | 559 | 171 | 121 | 779 | 324 | 329 | 311 | 3418 |
| Power-resources | 372 | 354 | 674 | 170 | 77 | 611 | 490 | 415 | 609 | 3363 |
| Security-personal | 82 | 240 | 83 | 150 | 110 | 195 | 81 | 107 | 519 | 1911 |
| Security-societal | 763 | 540 | 970 | 559 | 325 | 1059 | 425 | 798 | 947 | 6056 |
| Self-direction-action | 375 | 503 | 102 | 263 | 117 | 269 | 95 | 321 | 557 | 2567 |
| Self-direction-thought | 66 | 166 | 24 | 44 | 26 | 170 | 60 | 163 | 177 | 1265 |
| Stimulation | 324 | 314 | 221 | 60 | 81 | 150 | 429 | 49 | 320 | 1547 |
| Tradition | 138 | 91 | 49 | 87 | 32 | 196 | 28 | 52 | 336 | 1251 |
| Universalism-concern | 522 | 466 | 413 | 336 | 185 | 548 | 270 | 477 | 476 | 3667 |
| Universalism-nature | 191 | 239 | 189 | 125 | 98 | 160 | 244 | 219 | 196 | 1465 |
| Universalism-tolerance | 37 | 136 | 115 | 88 | 46 | 105 | 48 | 100 | 86 | 713 |
| Total | 5163 | 5492 | 5191 | 3346 | 1729 | 6093 | 4174 | 4318 | 7742 | 43248 |

# **Annex 5: Value Attainment**

| Value | Percentage of cases | | |
| --- | --- | --- | --- |
|  | Attained | Undecided | Constrained |
| Self-direction | 77.0 | 5.8 | 17.2 |
| Self-direction-thought | 77.5 | 7.6 | 14.9 |
| Self-direction-action | 76.7 | 5.2 | 18.1 |
| Stimulation | 82.3 | 7.1 | 10.5 |
| Hedonism | 70.7 | 3.6 | 25.7 |
| Achievement | 67.7 | 5.3 | 26.9 |
| Power | 60.7 | 11.9 | 28.1 |
| Power-dominance | 69.9 | 13.1 | 17.0 |
| Power-resources | 57.0 | 11.8 | 31.2 |
| Face | 47.1 | 8.0 | 44.9 |
| Security | 37.8 | 6.1 | 56.2 |
| Security-personal | 30.2 | 5.9 | 63.8 |
| Security-societal | 39.4 | 6.2 | 54.4 |
| Tradition | 79.1 | 6.7 | 14.2 |
| Conformity | 53.8 | 9.4 | 37.1 |
| Conformity-rules | 55.6 | 10.5 | 33.9 |
| Conformity-interpersonal | 41.4 | 5.3 | 53.3 |
| Humility | 73.5 | 2.2 | 24.3 |
| Benevolence | 80.1 | 5.3 | 14.7 |
| Benevolence-caring | 83.7 | 5.9 | 10.3 |
| Benevolence-dependability | 75.9 | 4.5 | 19.6 |
| Universalism | 62.3 | 5.3 | 32.7 |
| Universalism-concern | 63.8 | 4.9 | 31.3 |
| Universalism-nature | 65.7 | 6.0 | 28.3 |
| Universalism-tolerance | 47.9 | 5.3 | 46.8 |
|  |  |  |  |

*Note*. Percentage “attainment” level per fine grained value (10). Each value falls into “(partially) attained”, “(partially) constrained” and “Don’t know”.

# **Annex 6: An Assessment of Annotator Bias**

Because annotation tasks inevitably involve human judgment, best practice recommends explicitly auditing whether annotator characteristics systematically covary with annotation outcomes. Following this approach, we examine the association between annotators’ personal value priorities and the relative frequency of values annotated as a robustness check on the ValuesML annotation dataset. The aim of this supplemental analysis is to assess the independence of annotation decisions from annotators’ own value priorities under the constraints of a shared annotation guidelines and training procedures.

From a robustness perspective, the absence of systematic associations between annotators’ personal value priorities and annotation frequencies would indicate that value detection and interpretation is primarily driven by textual content and common annotation guidelines, consistent with the goal of producing an agnostic dataset. Conversely, the presence of systematic covariation would flag a potential source of annotator-related variance that may warrant further scrutiny or statistical control, without implying intentional projection or idiosyncratic bias at the individual level. This robustness check is designed to address concerns raised in annotation-based research.

First, personal values were measured independently of the annotation task and prior to analysis, reducing risks of circularity or post-hoc rationalization. Second, annotators operated within language-specific teams using a shared codebook, training materials, and adjudication procedures, placing some constraints on individual discretion. Third, analyses focus on relative frequencies of annotated values, minimizing confounding due to variation in annotation volume across annotators or language groups. Together, these design features align the analysis with established practices for assessing annotator-related variance in human annotated datasets.

To conduct this robustness check, we draw on survey data collected from annotators participating in the ValuesML project. In total, 84 annotators were recruited by the Joint Research Centre (JRC) via an open call requiring demonstrated expertise in values research and fluency in at least one of nine target languages (Bulgarian, Dutch, English, French, German, Greek, Hebrew, Turkish, and Italian). Annotators were assigned to language-specific teams and completed annotations on texts in the focal language. Of the 84 annotators recruited, 66 completed the annotation task. Full details of the number of annotators, texts, and annotations per language group are reported in Table A.

At the commencement of the ValuesML project, annotators were invited by the JRC to complete a brief online survey assessing their personal values and political attitudes. Ethics approval for this survey was granted by the [redacted for review]. In total, 59 annotators completed the survey (71% female).

**Method**

**Measures**

*Personal Values.* Annotators’ personal values were measured using the Portrait Values Questionnaire-Revised (PVQ-RR; Schwartz et al., 2012). The PVQ-RR comprises 57 items, with 3 items assessing each of the 19 refined values. Each item consists of a single sentence ‘portrait’ describing a gender-matched individual in terms of their goals or aspirations. Respondents indicate how similar they are to the person described using a 6-point scale (*1 = not like me at all, 6 = very much like me*). Example benevolence items include, “It is important to him/her/them to take care of people he/she/they are close to” and “It is important to him/her/them to be a dependable and trustworthy friend”. Scores for the 19 refined values and 10 basic values were computed following Schwartz’s (2016) procedure. Specifically, responses were averaged across items for each value and then mean-centred within respondents to control for individual differences in scale use and response tendencies.

*Annotations*. All texts, text spans, and value labels produced by annotators were extracted from INCEpTION in tab separated format. The annotation dataset included an anonymized annotator identifiers, language group, text identifiers, the value(s) assigned to each text span, and whether the value was annotated as attained, constrained, or uncertain. For analysis, the number of times each value was annotated was aggregated to produce per-annotator counts for each value. To account for differences in overall annotation volume, counts for each value were converted into relative frequencies by dividing the total number of annotations produced by each annotator. For those annotators who completed the personal values survey (*n* = 59), a total of 71,899 annotations were produced (*M* = 1,219, *SD* = 859).

**Analytic Approach**

To assess whether annotators’ personal value priorities covaried with the relative frequency of values annotated in text, Spearman’s rank-order correlations were computed between annotators’ value scores and their relative annotation frequencies for each refined value. This non-parametric approach was selected to accommodate skewed distributions and to align with the robustness-check framing of the analysis.

**Table A**

*Means and Standard Deviations for Annotators’ personal Values and value annotations*

| *Annotator values* |  |  | *Value annotations* |  |  |
| --- | --- | --- | --- | --- | --- |
| Basic Values | Mean | Std. Deviation | *basic* | Mean | Std. Deviation |
| SD10 | 5.42 | 0.50 | prSD10 | 0.09 | 0.05 |
| ST10 | 4.06 | 0.98 | prST10 | 0.04 | 0.05 |
| HE10 | 4.10 | 1.03 | prHE10 | 0.01 | 0.01 |
| AC10 | 4.34 | 0.93 | prAC10 | 0.10 | 0.05 |
| PO10 | 2.25 | 0.86 | prPO10 | 0.16 | 0.07 |
| SE10 | 4.16 | 1.02 | prSE10 | 0.21 | 0.07 |
| TR10 | 2.25 | 1.13 | prTR10 | 0.02 | 0.02 |
| CO10 | 3.66 | 1.13 | prCO10 | 0.12 | 0.04 |
| BE10 | 5.16 | 0.64 | prBE10 | 0.07 | 0.04 |
| UN10 | 5.06 | 0.60 | prUN10 | 0.14 | 0.06 |

**Results**

Consistent with the robustness-check framing outlined in the introduction, associations between annotators’ personal value priorities and the relative frequency with which values were annotated were generally weak, sparse, and unsystematic (see Table B). Across analyses, we did not observe the broad pattern of value-congruent associations that would be expected if annotators systematically projected their own value priorities onto the texts they annotated.

*All annotations (attained, constrained, and uncertain)*

When considering all annotations combined, only a small number of significant associations emerged. Power values were positively associated with the relative frequency of stimulation value annotations, while stimulation values were negatively associated with the relative frequency of power value annotations. Benevolence values were negatively associated with the relative frequency of self-direction annotations. Security and tradition values were negatively associated with the relative frequency of tradition annotations. Importantly, these associations did not form a consistent value-congruence pattern and included both positive and negative relations.

*Attained annotations only*

Analyses restricted to values annotated as *attained* revealed a similarly limited and mixed pattern. Power values were positively associated with the relative frequency of stimulation annotations, and tradition values were positively associated with the relative frequency of conformity annotations. In contrast, benevolence values were negatively associated with self-direction annotations, security values were negatively associated with hedonism annotations, and achievement values were negatively associated with security annotations. Additionally, security, conformity, and tradition values were all negatively associated with the relative frequency of tradition annotations. As with the full annotation set, these effects did not systematically align with annotators’ own value priorities.

*Constrained annotations only*

For values annotated as *constrained*, few associations were observed. Achievement values were positively associated with the relative frequency of stimulation annotations, and hedonism values were positively associated with the relative frequency of hedonism annotations. However, benevolence, security, and conformity values were negatively associated with the relative frequency of self-direction annotations, and security values were negatively associated with tradition annotations. Again, this pattern does not indicate consistent value-congruent detection.

Taken together, the results provide little evidence that annotators’ personal value hierarchies systematically influenced which values they identified in text. Rather than showing pervasive value-congruent annotation tendencies, observed associations were infrequent, directionally mixed, and often negative. This pattern is consistent with the expectation, outlined in the introduction, that annotation decisions were primarily constrained by textual content and shared annotation guidelines rather than annotators’ own value priorities. Overall, the absence of consistent, value-congruent associations between annotators’ personal value priorities and annotation frequencies indicates that the ValuesML annotations are unlikely to be systematically shaped by annotators’ own value hierarchies, supporting the robustness of the annotation process.

**Table B**

*All Annotations (i.e., values labelled attained, constrained, and undecided)*

|  | prSD10 | prST10 | prHE10 | prAC10 | prPO10 | prSE10 | prTR10 | prCO10 | prBE10 | prUN10 |
| --- | --- | --- | --- | --- | --- | --- | --- | --- | --- | --- |
| SD10 | -0.103 | -0.025 | 0.197 | 0.160 | 0.162 | -0.207 | 0.013 | 0.172 | 0.122 | -0.159 |
|  | 0.438 | 0.851 | 0.134 | 0.226 | 0.220 | 0.115 | 0.924 | 0.192 | 0.356 | 0.230 |
| ST10 | 0.053 | 0.175 | 0.165 | -0.103 | -.294^*^ | 0.097 | 0.173 | 0.004 | 0.210 | 0.042 |
|  | 0.688 | 0.186 | 0.212 | 0.439 | 0.024 | 0.463 | 0.190 | 0.977 | 0.110 | 0.749 |
| HE10 | 0.141 | 0.034 | 0.130 | -0.016 | -0.120 | -0.089 | 0.009 | 0.014 | 0.100 | 0.101 |
|  | 0.288 | 0.797 | 0.325 | 0.902 | 0.364 | 0.505 | 0.944 | 0.918 | 0.452 | 0.447 |
| AC10 | 0.026 | 0.213 | -0.034 | 0.195 | 0.041 | -0.221 | 0.094 | -0.215 | 0.165 | -0.021 |
|  | 0.846 | 0.105 | 0.796 | 0.139 | 0.756 | 0.092 | 0.481 | 0.102 | 0.211 | 0.875 |
| PO10 | 0.063 | .299^*^ | -0.166 | -0.002 | -0.151 | -0.021 | -0.029 | -0.006 | 0.084 | -0.153 |
|  | 0.638 | 0.022 | 0.208 | 0.990 | 0.253 | 0.875 | 0.830 | 0.965 | 0.526 | 0.248 |
| SE10 | -0.179 | -0.018 | -0.182 | -0.068 | 0.194 | 0.057 | -.321^*^ | 0.028 | 0.020 | 0.003 |
|  | 0.174 | 0.890 | 0.167 | 0.608 | 0.141 | 0.666 | 0.013 | 0.834 | 0.883 | 0.982 |
| TR10 | -0.019 | -0.002 | -0.179 | -0.102 | 0.078 | 0.059 | -.276^*^ | 0.217 | -0.074 | -0.032 |
|  | 0.886 | 0.988 | 0.175 | 0.443 | 0.556 | 0.656 | 0.034 | 0.098 | 0.580 | 0.812 |
| CO10 | -0.170 | 0.211 | -0.138 | 0.067 | -0.001 | -0.102 | -0.225 | 0.047 | 0.147 | -0.024 |
|  | 0.199 | 0.109 | 0.299 | 0.613 | 0.995 | 0.440 | 0.087 | 0.723 | 0.266 | 0.858 |
| BE10 | -.376^**^ | 0.013 | -0.180 | -0.012 | 0.220 | -0.090 | 0.075 | 0.035 | -0.047 | 0.185 |
|  | 0.003 | 0.924 | 0.173 | 0.930 | 0.094 | 0.497 | 0.571 | 0.792 | 0.725 | 0.161 |
| UN10 | -0.158 | -0.043 | 0.190 | 0.103 | 0.169 | -0.098 | 0.104 | -0.024 | -0.013 | -0.045 |
|  | 0.233 | 0.748 | 0.150 | 0.436 | 0.200 | 0.461 | 0.432 | 0.858 | 0.921 | 0.736 |

*Note.* *N* = 59. Correlations are based on all annotations. Text in purple in tables denotes where the % of annotations for a value is less than 5% of the total.

**References**

Schwartz, S. H. (2016). Coding and analyzing PVQ-RR data (instructions for the revised Portrait Values Questionnaire). <https://www.researchgate.net/publication/308166496_Coding_and_analyzing_PVQ-RR_data_instructions_for_the_revised_Portrait_Values_Questionnaire>. DOI:[10.13140/RG.2.2.35393.56165](http://dx.doi.org/10.13140/RG.2.2.35393.56165)

**Annex 7: Word clouds of most used words in annotated value spans**
